# Supplementary material for: Pre-Incisional and Multiple Intradermal Injection of N-Acetylcysteine Slightly Improves Incisional Wound Healing in an Animal Model
Source: Int J Mol Sci. 2024 May 10;25(10):5200. doi: 10.3390/ijms25105200 (PMC11121603; doi:10.3390/ijms25105200)
Supplement: Supplementary file 1 [file ijms-25-05200-s001.zip › ijms-2974340-supplementary.pdf]

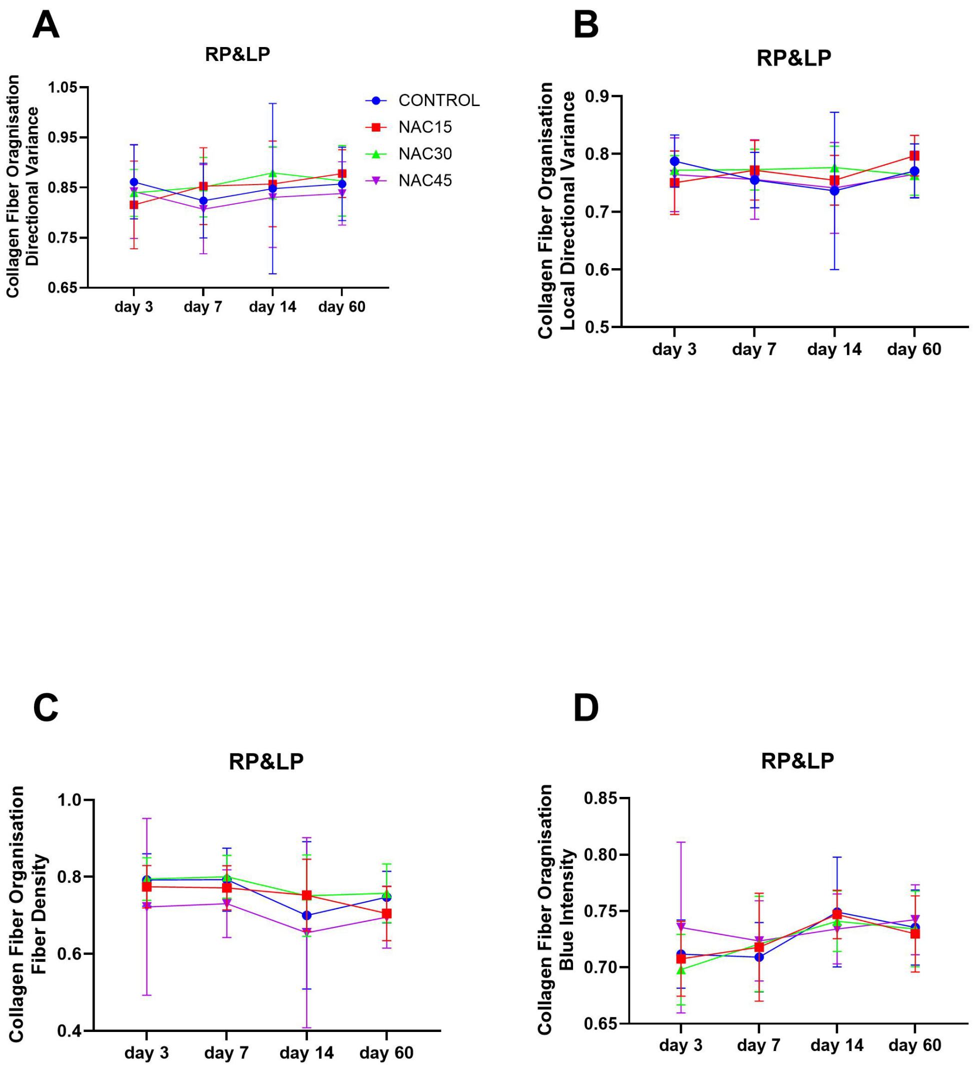

Figure S1. Graphs depicting directional variance (**A**), local directional variance (**B**), fiber density (**C**) and blue intensity (**D**) of collagen fibers on TM-stained sections collected from proximal scar areas at 3rd, 7th, 14th and 60th days. Values expressed as mean ± SD.

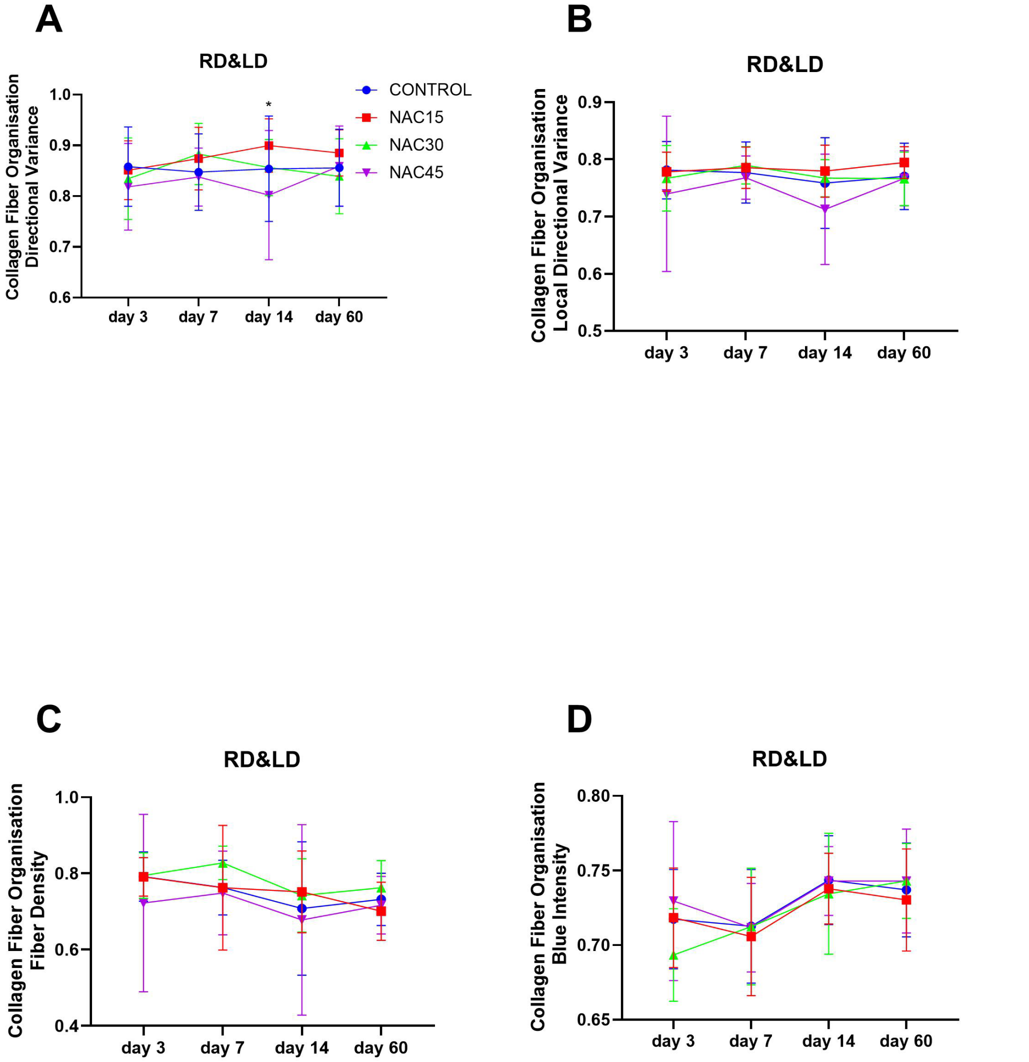

Figure S2. Graphs depicting directional variance (**A**), local directional variance (**B**), fiber density (**C**) and blue intensity (**D**) of collagen fibers on TM-stained sections collected from distal scar areas at 3rd, 7th, 14th and 60th days. Values expressed as mean ± SD. * - T-test, $p<0.05$ for NAC15 vs. NAC45.

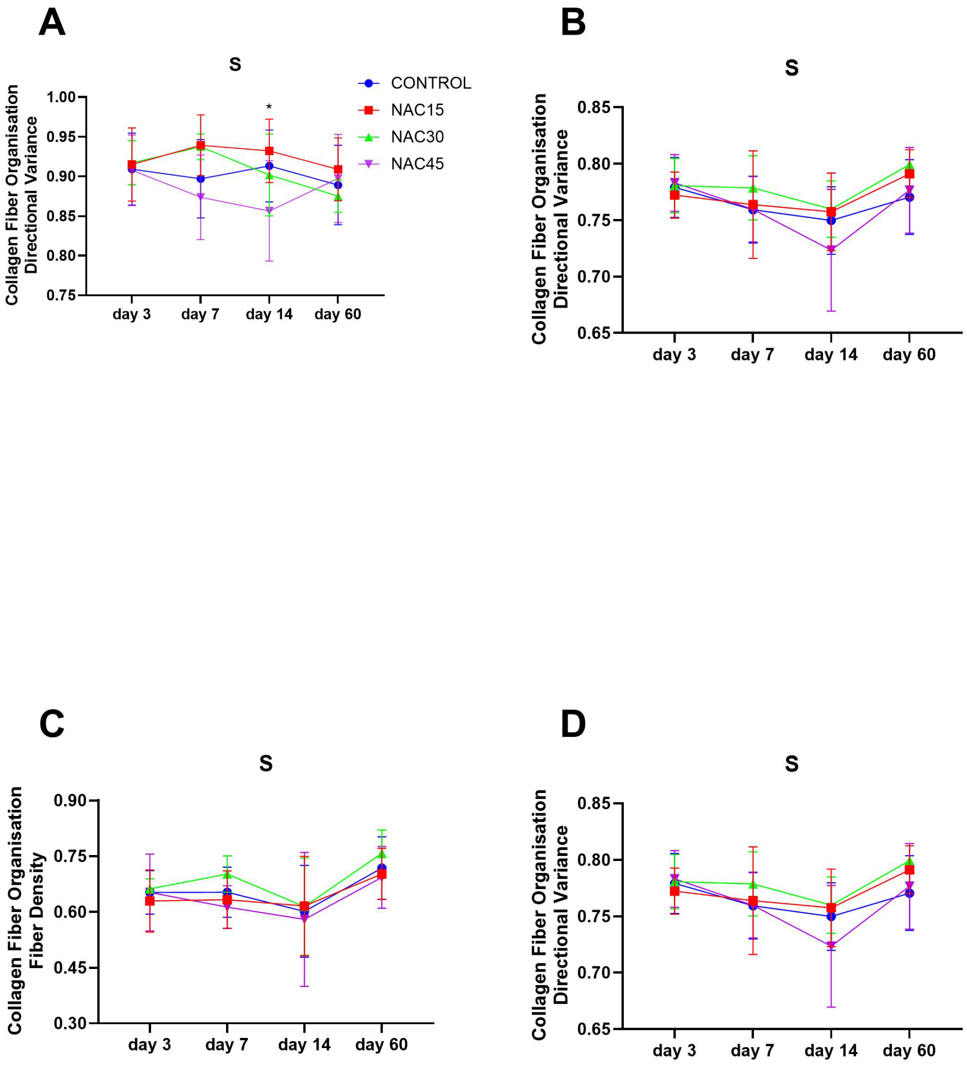

Figure S3. Graphs depicting directional variance (**A**), local directional variance (**B**), fiber density (**C**) and blue intensity (**D**) of collagen fibers on TM-stained sections collected from scar areas at 3rd, 7th, 14th and 60th days. Values expressed as mean ± SD. * - T-test, p<0.05 for NAC15 vs. NAC45.

Variable	Score			
	0	1	2	3
Acute inflammation	None	Scant	Moderate	Abundant
Chronic inflammation	None	Scant	Moderate	Abundant
Granulation tissue amount	None	Scant	Moderate	Abundant
Granulation tissue fibroblast maturation	Immature	Mild maturation	Moderate maturation	Fully matured
Collagen deposition	None	Scant	Moderate	Abundant
Reepithelization	None	Partial	Complete but immature or thin	Complete and mature
Neovascularization	None	Up to five vessels per HPF	6-10 vessels per HPF	More than 10 vessels per HPF

Table S1. Table depicting scoring for Histological Assessment of Scars using Abramov scale assesment. HPF - high power field.

Variable	Ranges of score	
	0	10
Collagen fiber orientation regularity	Uniform/regular	Chaotic/irregular
Collagen fiber density	Homogenous	Heterogenous
Collagen fiber diameter	Uniform/regular	Chaotic/irregular
Collagen fiber maturity	Old/ mature fibers	New/ fresh deposits

Table S2. Table depicting scoring for Histological Assessment of Scars using Detailed collagen fiber assesment.

Variable	Ranges of score	
	0	10
Overall collagen fiber assessment	Unaffected collagen fiber setting – corresponds to normal skin section. Regular fiber orientation, uniform fiber diameter	Chaotic collagen fiber distribution, deposits of a new collagen, variable fiber orientation, diameter, and density

Table S3. Table depicting scoring for Histological Assessment of Scars using Global collagen fiber assesment.

	Score			
Variable	0	1	2	3
Granulation tissue amount	None	Scant	Moderate	Abundant
Collagen deposition	None	Scant	Moderate	Abundant

Table S4. Table depicting scoring for Histological Assessment of Scars using collagen deposit and granulation tissue asssessment according to Abramov scale.

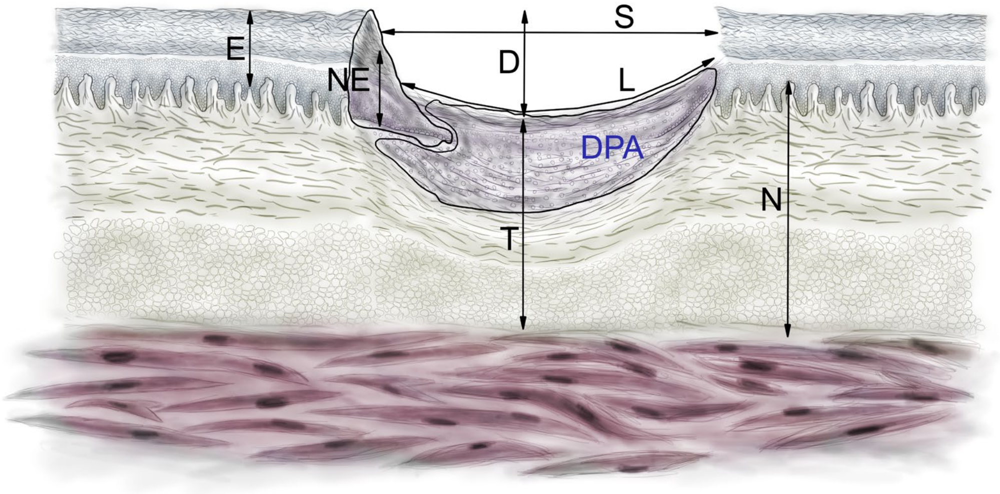

Figure S4. Diagram showing parameters measured in each HE section according to mathematical model of wound healing proposed by Lemo et al. (Vet. Arhiv 80 (5), 637-652, 2010) (for more information please visit DOI - 10.3390/ijms22147549 **).** D-depth of the wound, DPA -dermal proliferation area, E-thickness of the epidermis, L-length of the re-epithalization zone, N-thickness of the natural dermis, NE-thickness of newly formed epidermis, S-distance between borders of the wound, T-thickness of the connective tissue in the wound. (diagram taken from DOI - 10.3390/ijms22147549)

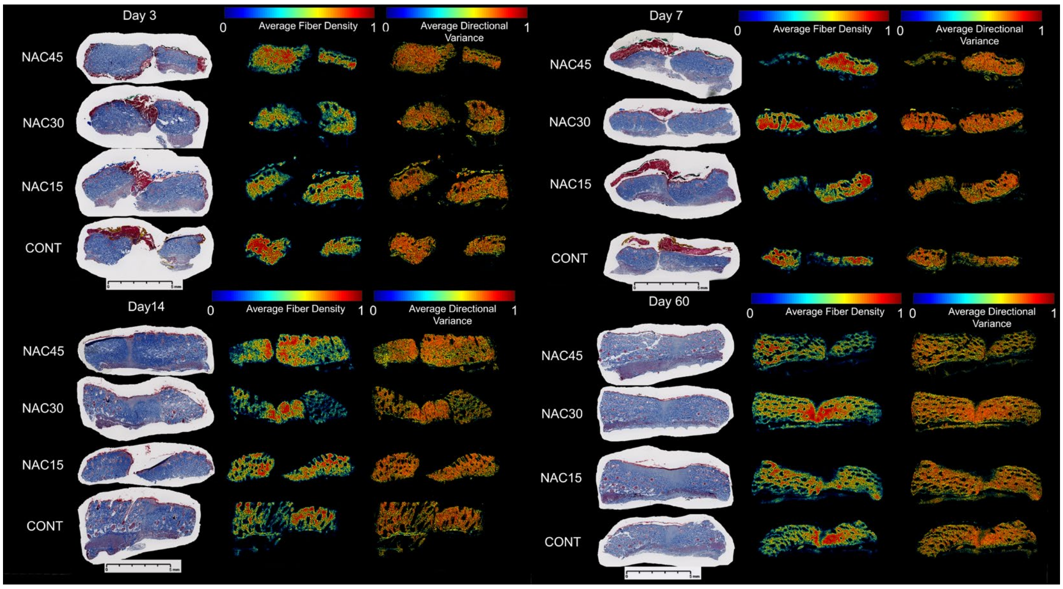

Figure S5. Representative results of automated collagen fiber arrangement analysis by Quinn et al. Heatmaps of collagen and fiber density and average directional variance are presented along with Trichome Masson's staining of each studied group (NAC15, NAC30, NAC45) in four harvest days post op: 3rd, 4th, 14th and 60th. Statistical analysis showed statistically significant decrease in directional variance of collagen fibers in the NAC45 group compared to NAC15 in distal scar areas (RD&LD) and the scar (S) at day 14 post-op. None of the rest of the values were statistically significant (for more information please visit DOI - 10.3390/ijms22147549 or into table S6) (graphic taken from DOI - 10.3390/ijms22147549)

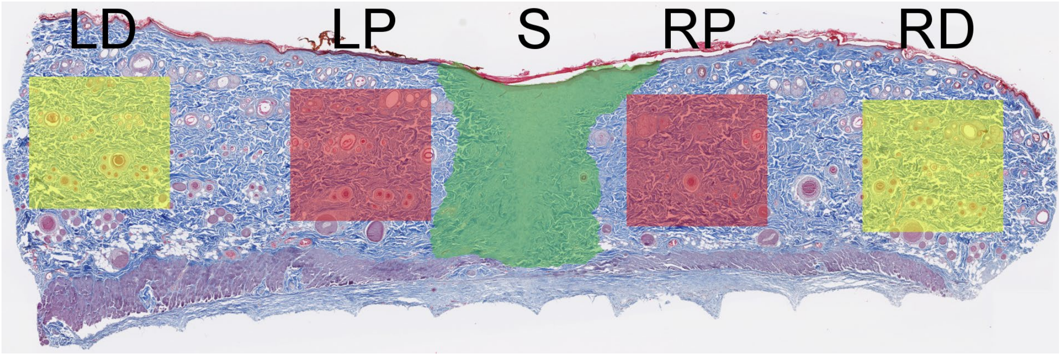

Figure S6. Diagram representing regions of the wound used for automated collagen fiber analysis in MT-stained samples. Five areas were chosen, scar and four boxes 500 by 500 pixels of proximal and distal areas from the scar area. LD-left distant area, RD-right distant area, LP-left proximal area, RP-right proximal area, S-scar zone (for more information please visit DOI - 10.3390/ijms22147549) (diagram taken from DOI - 10.3390/ijms22147549)

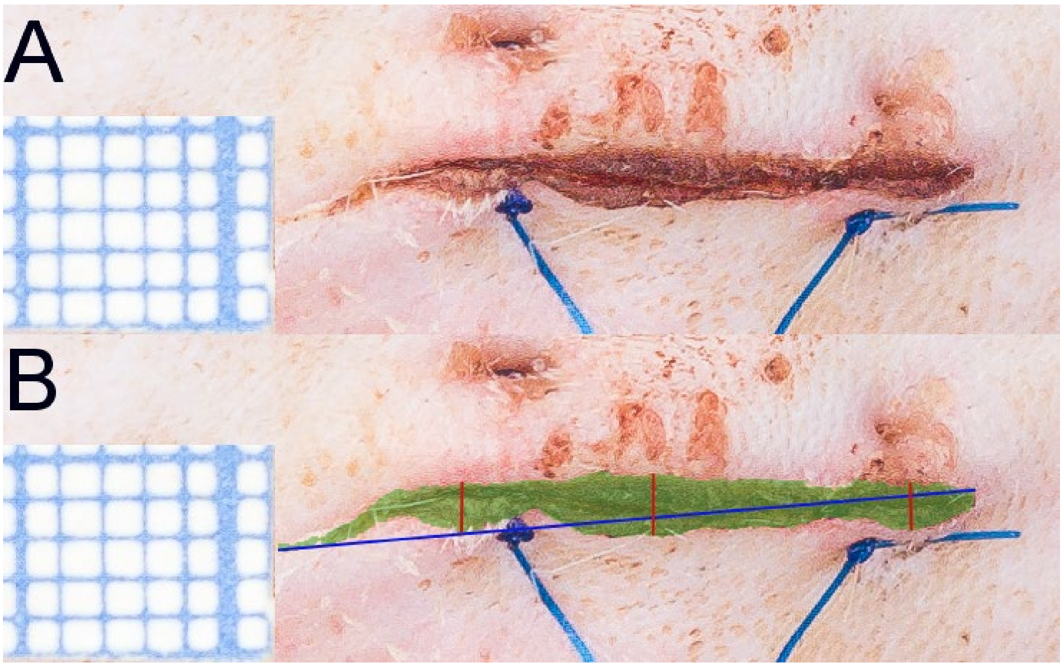

Figure S7. (**A**) A 3rd day wound post-op with a surgical microscale (1sqr-1mm), (**B**) wound with marked surface area (green zone), length (blue line) and width (red lines) by ImageJ by a blinded researcher. (for more information please visit DOI - 10.3390/ijms22147549) (graphic taken from DOI - 10.3390/ijms22147549)

Table S5: Raw data of histomorphometrical analysis

Rat no,	Wound no,	Injection	Harvest day	Experimental group (C/A15/A30/A45)	Control for (A15/A30/A45 or c_A15/c_A30/c_A45)	Gross division	D1	D2	DPA	EPI	L (perimeter)	N	NEO	S	T	H	B	Lost	SCI (superficial contraction index)	DCI (deep contraction index)	WSI (wound severity index)	GHI (global healing index)	GCI (global contraction index)	HRI (hair remodeling index)	MRI (matrix remodeling index)	GRI (global remodeling index)
1	A	M	7	A45	A45	A	179,15	284,81	46085	43	1654,2	909,09	85,8	630,22	747,68	240,98	20040,6	1023,98	0,384538761	0,802934803	0,177551178	1,009922386	1,187473563	0,89710249	0,768332538	0,832717514
1	B	M	7	A30	A30	A	406,72	447,37	21859,1	52,3	2576,2	1195	73,02	1222,5	925,61	2622,5	17744,2	1353,7	0,096919554	0,659648536	0,225430962	0,531137127	0,756568089	-0,11979716	0,794878707	0,337540774
1	C	M	7	A15	A15	A										3502,9	21028,1							-0,495724488	0,75691713	0,130596321
1	E	M	7	C	c_A30	C	481,13	578,19	28441,4	48,5	3722	1193	57	1710,6	959,76	1939,8	26115,8	2011,4	0,149547579	0,596705784	0,195507125	0,550746238	0,746253363	0,171713048	0,698103794	0,434908421
1	F	M	7	C	c_A15	C	332,47	356,16	44104	57,04	4329,5	1266	82,2	1747	375,53	2439,7	32988,3	2582,5	0,323523717	0,737385466	0,703372828	0,357536356	1,060909183	-0,041742281	0,618658336	0,288458028
5	A	M	60	C	c_A45	C	123,13	141,92	163934	33	1818,5	1309,2	40,02	614,51	1235,7	643,24	556737	1203,99	0,489605395	0,905950199	0,056141155	1,339414439	1,395555594	0,725339056	-5,435827664	-2,355244304
5	B	M	60	C	c_A15	C	88,23	118,08	810537	34	860,55	1657,3	64,01	397,82	1981			462,73	0,140276187	0,946762807	-0,195317685	1,282356679	1,087038994			
5	C	M	60	C	c_A30	C	134,64	147,32	1242729	28,2	1055,8	1757,23	49,22	496,22	2204,7	2188,3	1880000	559,58	0,113227778	0,92337941	-0,254645095	1,291252282	1,036607188	0,065604528	-20,73262422	-10,33350984
5	D	M	60	A45	A45	A	182,89	400,95	892683,5	25,87	2109	1789,6	54,29	983,22	1599,2	1222,9	1072222,9	1125,78	0,126632202	0,897803979	0,10639249	0,91804369	1,02443618	0,477826522	-11,39479647	-5,458484974
5	E	M	60	A15	A15	A	110,67	278,49	3664293	23,8	1716,9	1853,35	48,61	831,09	1639,3	1580,9	3539164	885,81	0,06177397	0,940286508	0,115493566	0,886566912	1,002060478	0,324961933	-39,91240492	-19,7937215
5	F	M	60	A30	A30	A	306,12	307,06	834867	30,41	3051,5	1961,58	49,59	1475,5	1759,6	1461,4	1147104	1576	0,063769036	0,843942128	0,102968016	0,804743148	0,907711164	0,375987962	-12,26041498	-5,942213511
11	A	M	14	A45	A45	A	874,13	914,08	3082549,5	54,2	7426,8	1275	108,6	3426,4	1149,6	3033,4	3665001	4000,4	0,143485651	0,314407843	0,098352941	0,359540553	0,457893495	-0,29524984	-41,36706888	-20,83115936
11	B	M	14	A15	A15	A	492,65	891,18	655287	71,5	6482,7	1609,4	135,17	3105,9	1332,4	5595,9	1659885	3376,8	0,080223881	0,693892134	0,172113831	0,602002183	0,774116014	-1,389427236	-18,1881154	-9,788771317
11	C	M	14	A30	A30	A										4799,7	2311309							-1,049452975	-25,71851593	-13,38398445
11	D	M	14	C	c_A45	C	484,56	480,33	2563256,7	83,1	2836,1	1445,8	97,12	1316,6	1267,1	5382,7	4190487	1519,5	0,133530767	0,66484991	0,123599391	0,674781285	0,798380677	-1,298391677	-47,4416379	-24,37001479
11	E	M	14	C	c_A15	C	261,42		633977,8	36,02	4329,3	1518,65	95,7	2095,5	1038,3	1791,2	1040650	2233,8	0,061912436	0,827860271	0,316300662	0,573472045	0,889772707	0,235164662	-11,0298167	-5,397326018
11	F	M	14	C	c_A30	C										4495,3	1323454							-0,919475376	-14,2990045	-7,609239936
13	A	M	7	A45	A45	A	325,88	420,77	496319,2	60,57	1941,4	793,32	131,35	875,03	581,73	4897	459902	1066,37	0,179431154	0,589219987	0,266714567	0,501936575	0,768651141	-1,090999692	-4,316424119	-2,703711905
13	B	M	7	A30	A30	A	383,9	457,4	19768,2	48,15	2609,6	1335,64	125,21	1236,6	888,68	2928,5	21208	1373	0,099344501	0,71257225	0,334641071	0,47727568	0,811916751	-0,250457953	0,754837503	0,252189775
13	C	M	7	A15	A15	A										4870,5	407903							-1,079684296	-3,715320541	-2,397502418
13	D	M	7	C	c_A45	C										2709,9	95072							-0,157116615	-0,099023431	-0,128070023
13	E	M	7	C	c_A30	C										1583,6	210489							0,323809044	-1,433233159	-0,554712058
15	A	M	14	A30	A30	A	703,58	1463,5	1047721	45,74	9352,7	2041,9	123,7	4319,8	1911,2	2990,7	2389040	5032,9	0,141687695	0,655428767	0,064009011	0,733107451	0,797116462	-0,277017108	-26,61707902	-13,44704807

15	B	M	14	A45	A45	A	600,577	779,07	560145	70,26	3361,5	2078,2	140,61	1489,1	2292,8	4181,3	1306086	1872,4	0,204710532	0,711014339	-0,103262439	1,01898731	0,915724871	-0,785398614	-14,09823204	-7,441815327
15	D	M	14	C	c_A30	C	352,91	357,01	680135,9	44,2	1060,6	2529,5	221,49	334,86	2073,5	908,78	880930	725,74	0,538595089	0,860482309	0,180272781	1,218804617	1,399077398	0,611954523	-9,183468432	-4,285756954
15	E	M	14	C	c_A45	C	258,66	300,44	415829,9	77,17	1615	2450,73	250,77	722,69	2213,8	3504,3	1164400	892,31	0,190090888	0,894455938	0,096677317	0,987869509	1,084546825	-0,496322283	-12,46035513	-6,478338705
15	F	M	14	C	c_A15	C	240,65	322,62	304755	39,58	1522	2166,4	55,07	716	1832,9	93,14	331736	806	0,111662531	0,888917097	0,153942024	0,846637605	1,000579629	0,960229587	-2,834837142	-0,937303778
18	A	M	60	C	c_A45	C	0	0	241820,5	33,894	0	1424	27,61	0	1288	304,84	2689492			1	0,095505618	0,904494382	1	0,869834522	-30,09027605	-14,61022076
18	B	M	60	C	c_A15	C	56,68	591,96	827200,5	27,69	3704,1	1571,76	29,97	1830,4	1566,5	869,87	1103283	1873,7	0,023109356	0,963938515	0,003346567	0,983701304	0,987047871	0,62856894	-11,75384832	-5,562639691
18	C	M	60	C	c_A30	C	208,12	198,33	1439836,5	40,34	1747,1	1486,42	94,83	821,75	1297,8	1581,9	2212823	925,35	0,111957638	0,859985738	0,126895494	0,845047881	0,971943375	0,324534937	-24,58002698	-12,12774602
18	D	M	60	A45	A45	A	137,82	303,28	473999	27,26	2010,1	1490,57	25,24	993,54	1355,9	488,74	342406	1016,56	0,022644999	0,907538727	0,090347988	0,839835738	0,930183726	0,791309947	-2,958181345	-1,083435699
18	E	M	60	A15	A15	A										1277,9	1395018							0,454341739	-15,12627764	-7,335967953
18	F	M	60	A30	A30	A	241,35	735,5	3155580,3	20,02	5000,6	1791,85	117,39	2476,7	1095,2	4033	3688859	2523,9	0,018701216	0,865306806	0,388788124	0,495219898	0,884008022	-0,722075098	-41,64286513	-21,18247011
20	A	M	14	A15	A15	A	216,75	266,51	416342	39,47	1627,1	1188,17	105,96	739,35	1221,4	1562,3	1131525	887,75	0,167164179	0,817576609	-0,027967378	1,012708167	0,984740789	0,33290406	-12,0803232	-5,873709571
20	B	M	14	A45	A45	A	677,64	730,62	924675	53,68	3584,1	1779,27	111,4	1532,6	1124,9	1296	458566	2051,5	0,252936875	0,619147178	0,367774424	0,504309629	0,872084054	0,44661311	-4,300980084	-1,927183487
20	C	M	14	A30	A30	A	337,49	681,3	250378	48,69	3727,8	1599,7	82,13	1808,6	1348,6		443106	1919,2	0,057628178	0,789029193	0,156966931	0,68969044	0,846657371		-4,122263929	-2,061131965
20	D	M	14	C	c_A15	C	210,95	214,82	557661	34,28	3197,2	1301,4	59,67	1238,3	1507,6	1084,3	699820	1958,9	0,367859513	0,837905333	-0,158444752	1,364209598	1,205764846	0,537008175	-7,089853766	-3,276422795
20	E	M	14	C	c_A45	C	149,97	259,08	446727,7	38,156	2549	1580,3	54,74	1257	1506,3	2592,6	2116695	1292	0,027089783	0,905100297	0,046826552	0,885363529	0,932190081	-0,107029978	-23,46879629	-11,78791313
20	F	M	14	C	c_A30	C	194,96	468,18	993442	90,22	1900	1255,4	147,07	927	1155,4	8521,2	1086877	973	0,047276465	0,844702884	0,079655887	0,812323462	0,891979348	-2,638518802	-11,5641965	-7,101357649
22	A	M	60	C	c_A15	C	262,62	386,53	401052,4	25,615	3024	1186,4	36,71	1397,5	1347	532,81	238301	1626,5	0,140793114	0,778641268	-0,135367498	1,05480188	0,919434382	0,772492231	-1,754737279	-0,491122524
22	B	M	60	C	c_A45	C	410,39	568,27	2590095,3	25,762	7523,9	1443,07	35,85	3737,7	1573,1	2227	1857310	3786,2	0,012809677	0,715613241	-0,090106509	0,818529428	0,728422918	0,049079781	-20,47032994	-10,21062508
22	C	M	60	C	c_A30	C	101,33	218,78	1231981,9	31,176	536	1788,1	60,9	254	1983,5	494,28	1121421	282	0,09929078	0,94333091	-0,109278005	1,151899695	1,04262169	0,788944389	-11,96352191	-5,587288758
22	E	M	60	A45	A45	A										855,17	959470							0,634845782	-10,09138349	-4,728268853
22	F	M	60	A30	A30	A	288,82	609,24	1812253	24,839	3570,1	1656,73	49,465	1637,5	1582,5	1587,8	1091399	1932,6	0,15269585	0,825668636	0,044805128	0,933559358	0,978364487	0,322015661	-11,61647039	-5,647227366
27	A	M	60	C	c_A15	C	117,44	115,23	626672	32,31	1021	1890,83	44,67	479,41	1694,4	528,99	771857	541,59	0,114810096	0,93788971	0,103885595	0,948814211	1,052699806	0,774123356	-7,92259475	-3,574235697
27	B	M	60	C	c_A45	C	731,29	783,79	3020247	33,41	8827,1	2006,75	180,18	4101,6	1837,9	2481,3	3124830	4725,5	0,132028357	0,635584901	0,084141024	0,683472234	0,767613258	-0,059505317	-35,12274262	-17,59112397
27	C	M	60	C	c_A30	C	493,55	1101,5	5324310	54,264	6979,6	1856,48	110,87	3259,2	1716	4939,5	6197540	3720,4	0,123965165	0,734147419	0,075670085	0,782442498	0,858112584	-1,109147024	-70,64298292	-35,87606497
27	D	M	60	A15	A15	A	221,73	238,87	768415,5	30,13	1752	1825,53	81,1	684,57	2111	621,37	1718362	1067,43	0,358674574	0,878539383	-0,156376504	1,393590461	1,237213957	0,73467746	-18,86410405	-9,064713296

27	E	M	60	A45	A45	A	264,5	334,58	1678805	66,08	1876,1	1570,21	127,84	890,6	1533,4	2297,1	2272372	985,5	0,096296296	0,831551194	0,023442724	0,904404766	0,92784749	0,019147357	-25,26840785	-12,62463025
27	F	M	60	A30	A30	A	299,01	304,75	2516862	32,34	2924	1866,3	111,03	1325,1	1185,4	4056,4	2831566	1598,9	0,171242729	0,839784601	0,364839522	0,646187808	1,01102733	-0,732066806	-31,73263821	-16,23235251
28	A	M	14	A30	A30	A										4773,6	3105907							-1,038308378	-34,90399451	-17,97115145
28	B	M	14	A15	A15	A	179,6	207,43	346044	42,49	1278	1654,07	87,53	628,06	1286,6	2014,1	470672	649,94	0,033664646	0,891419347	0,222161094	0,7029229	0,925083993	0,139987241	-4,440924312	-2,150468535
28	C	M	14	A45	A45	A	164,26	166,98	425362,7	43,781	847,94	1437,2	101,2	405,51	1360	1521,5	472811,5	442,43	0,083448229	0,885708322	0,053715558	0,915440993	0,969156551	0,350325499	-4,465656732	-2,057665616
28	D	M	14	C	c_A30	C	304,58	306,32	471587,3	55,149	3476,1	1098,945	99,32	1581,9	878,17	3047,7	898569	1894,2	0,164871714	0,722843272	0,200897224	0,686817762	0,887714986	-0,301355883	-9,387373623	-4,844364753
28	E	M	14	C	c_A15	C	121,05	249,67	305776,9	46,41	1738	1772,75	64,49	854,23	1844,8	3776,4	641303	883,77	0,033424986	0,93171626	-0,040643069	1,005784315	0,965141246	-0,61250791	-6,413402717	-3,512955314
28	F	M	14	C	c_A45	C	292,89	294,27	192574,7	88,69	1092,9	1261,28	121,17	383,13	992,7	5533,7	879973	709,77	0,460205419	0,767783522	0,212942408	1,015046532	1,22798894	-1,362868081	-9,172405602	-5,267636841
29	A	M	7	A15	A15	A	408,23	416,98	25326,2	37,345	1927	798,09	117,7	894,12	786,58	3506,4	360951,6	1032,88	0,134342808	0,488491273	0,014421932	0,608412149	0,622834081	-0,497218975	-3,172566747	-1,834892861
30	B	M	7	A15	A15	A	452,24	535,85	80098,9	50,728	3203,5	1291,77	110,28	1458,7	570,44	2976,8	26759,2	1744,8	0,163972948	0,649906717	0,55840436	0,255475305	0,813879665	-0,271081863	0,69066615	0,209792144
30	C	M	7	A30	A30	A	623,81	622,49	22199	53,358	4641,9	1332,1	55,59	2225,9	460,67	2518,5	1234,9	2416	0,078683775	0,531709331	0,654177614	-0,043784508	0,610393106	-0,07538957	0,985724671	0,455167551
30	D	M	7	C	c_A45	C	525,27	535,68	95195,9	50,817	3223,5	1164,95	117,7	1355,8	1063,7	3736,8	51282,7	1867,7	0,27408042	0,549105112	0,086913601	0,73627193	0,823185532	-0,595598866	0,407176783	-0,094211042
30	E	M	7	C	c_A15	C	328,98	366,02	105299	42,351	3293,9	1288,85	59,48	1629,1	937,27	3176,6	96410,1	1664,8	0,021444017	0,744749195	0,272785817	0,493407396	0,766193212	-0,356395675	-0,114491742	-0,235443708
30	F	M	7	C	c_A30	C	880	966,95	37022	43,523	5382,9	1273,35	59,22	2342,7	603,44	2246,3	40469,3	3040,2	0,229425696	0,308909569	0,526100444	0,012234821	0,538335265	0,040838757	0,532178676	0,286508716
33	A	M	60	A45	A45	A	97,67	95,05	379141,7	33,3	716,68	1405,67	39,1	353,03	1188	508,61	574130	363,65	0,029203905	0,93051712	0,154851423	0,804869602	0,959721025	0,782825535	-5,636889118	-2,427031791
33	B	M	60	A15	A15	A	174,44	243,82	3104943,3	65	1753	1553,25	97,8	849	1671	979,7	2364125	904	0,060840708	0,887693546	-0,075808788	1,024343042	0,948534254	0,581671963	-26,32906395	-12,87369599
33	C	M	60	A30	A30	A	262,21	754,8	2303438,5	48,672	4436,9	1557,03	72,18	2153,6	1348,8	3701,6	3139012	2283,3	0,056803749	0,831596051	0,133735381	0,75466442	0,8883998	-0,580568605	-35,28668522	-17,93362691
33	D	M	60	C	c_A45	C	162,51	207,54	559123,3	30,378	1377,7	1378,79	49,832	580,18	1450,6	852,2	775974	797,52	0,272519811	0,882135786	-0,052081898	1,206737495	1,154655597	0,63611396	-7,970186886	-3,667036463
33	E	M	60	C	c_A15	C	175,16	220,74	2020224	32,204	1586	1561,78	138,9	758,97	1265,2	2479,9	2570246	827,03	0,082294475	0,887845919	0,189898705	0,780241689	0,970140395	-0,058907522	-28,71180344	-14,38535548
33	F	M	60	C	c_A30	C	290,17	302,01	2363936,9	26,355	6824,6	1637,85	87,84	3219,9	1321,8	4110,2	3263191	3604,7	0,106749521	0,822834814	0,192966389	0,736617947	0,929584335	-0,755039194	-36,72218285	-18,73861102
35	A	M	14	A30	A30	A	255,04	280,01	405684	52,291	1957,1	947,69	112,73	905,96	874,49	2591,8	636942	1051,14	0,138116711	0,730882462	0,077240448	0,791758725	0,868999173	-0,106688381	-6,362989965	-3,234839173
35	C	M	14	A15	A15	A										4461,2	4990304,9							-0,90491481	-56,68745804	-28,79618643
35	E	M	14	C	c_A45	C	354,23	356,99	686253,8	63,49	3288,4	1180,7	118,49	1577,8	1389,3	6162,8	1138420	1710,6	0,077633579	0,699983061	-0,176674854	0,954291494	0,77761664	-1,631491301	-12,16002876	-6,895760029
35	F	M	14	C	c_A15	C	324,23	361,7	240652,3	37,828	4247	1557,38	82,45	2099,9	1547,3	913,52	354964	2147,1	0,02198314	0,791810605	0,006472409	0,807321336	0,813793745	0,609930562	-3,10335065	-1,246710044
37	A	M	14	C	c_A30	C	703,18	704,66	593842,7	40,52	3705,6	1318,92	140,16	1380	962,28			2325,6	0,406604747	0,466851667	0,270403057	0,603053357	0,873456414			

37	B	M	14	C	c_A15	C	446,17	444,7	228111,4	30,906	2615,6	1421,67	141,78	1202	1059,6	874,2	370374	1413,6	0,149688738	0,686164862	0,254679356	0,581174244	0,8358536	0,626720047	-3,28148881	-1,327384381
37	D	M	14	A30	A30	A	296,76	323,89	351784,1	33,226	2226	1227,4	133,63	921,48	1209,4	718,45	1053567	1304,52	0,293625241	0,758220629	0,014665146	1,037180725	1,05184587	0,693224683	-11,17913601	-5,242955664
37	E	M	14	A15	A15	A	305,71	315,11	269588,8	28,779	2800,3	1624,27	59,95	1347,2	1405,6			1453,1	0,072878673	0,811786218	0,134626632	0,750038259	0,884664891			
46	A	M	7	C	c_A45	C	529,09	568,83	215506,7	47,885	2813,7	1209,57	106,54	1215,7	982,42	1257,9	226017	1598	0,239236546	0,56258009	0,187794009	0,614022627	0,801816636	0,46288166	-1,612735387	-0,574926864
46	D	M	7	A45	A45	A	616,82	754,65	95389,5	50,436	4243,1	1197,58	170,64	1763	1147,4	1637,1	97190,5	2480,1	0,289141567	0,484944638	0,041901167	0,732185038	0,774086205	0,300964755	-0,123513093	0,088725831
47	A	M	60	C	c_A15	C	267,98	612,18	625951,8	26	3416,6	1320,57	29,84	1590	990,08	264,75	434628	1826,6	0,129530275	0,797072476	0,250263144	0,676339607	0,926602751	0,886952794	-4,024259042	-1,568653124
47	B	M	60	C	c_A30	C	37,46	153,43	764494,9	25,9	969,01	1931,27	45,78	430,47	1657	426,77	773646	538,54	0,200672188	0,980603437	0,142015358	1,039260267	1,181275625	0,817770893	-7,943275423	-3,562752265
47	C	M	60	C	c_A45	C	78,31	89,96	3245413	32,686	1392	1820,74	40,75	689,11	1418	1522,9	2911378	702,89	0,019604775	0,956990015	0,221195778	0,755399012	0,97659479	0,349727705	-32,65525746	-16,15276488
47	D	M	60	A15	A15	A	30,66	41,17	477686,3	21,053	483,46	1261,25	18,4	239,01	1326,8	775	444119	244,45	0,02225404	0,975690783	-0,05197225	1,049917072	0,997944823	0,669078056	-4,133974114	-1,732448029
47	E	M	60	A30	A30	A	75,72	127,06	1489618,9	27,151	1726,6	1552,08	36,37	837,59	1368,9	1152	994019	889,01	0,057839619	0,951213855	0,118022267	0,891031207	1,009053474	0,508100542	-10,4907667	-4,991333078
47	F	M	60	A45	A45	A	0	0	3812440,5	31,345	0	1569,65	72	0	1764,5	1612	3009429			1	-0,124135954	1,124135954	1	0,311682356	-33,78871785	-16,73851775

Table S6: Raw data of automated collagen fiber properties analysis

lp	Rat No	Wound No	Injection (S/M)	Experimental group (C/A15/A30/A45)	Control for (A15/A30/A45 or c_A15/c_A30/c_A45)	Harvest day	Gross division (A/C)		Name	Wound	Treatment	Location (D – distal, P – Proxiaml, L – left, R - right	Averaged	Wound /non wound	Average Directional Variance	Average Local Directional Variance	Average Fiber Density	Average Blue Intensity	Standard Deviation of Blue Intensity
1	1	A	M	A45	A45		7	A	1	A	A45	LD	MeanL	Blank	0,839256287	0,792402506	0,792344034	0,69690651	0,089291795
2	1	A	M	A45	A45		7	A	1	A	A45	LP	Blank	Blank	0,680269957	0,637444079	0,537445903	0,71489136	0,072495719
3	1	A	M	A45	A45		7	A	1	A	A45	RD	MeanR	Blank	0,844926476	0,801101029	0,867373884	0,756895785	0,071142376
4	1	A	M	A45	A45		7	A	1	A	A45	RP	Blank	Blank	0,886409998	0,813510299	0,690080941	0,803733239	0,061826114
5	1	A	M	A45	A45		7	A	1	A	A45	Wound	Wound	whole	0,900175333	0,792158663	0,568498492	0,739547697	0,064170874
6	1	B	M	A30	A30		7	A	1	B	A30	LD	MeanL	Blank	0,928044379	0,804447353	0,841669321	0,737705375	0,073899412
7	1	B	M	A30	A30		7	A	1	B	A30	LP	Blank	Blank	0,878975749	0,802976072	0,85800457	0,740806808	0,068508949
8	1	B	M	A30	A30		7	A	1	B	A30	RD	MeanR	Blank	0,927379966	0,787204504	0,871300697	0,749836544	0,074096251
9	1	B	M	A30	A30		7	A	1	B	A30	RP	Blank	Blank	0,729833841	0,688056231	0,741790891	0,755803873	0,08089384
10	1	B	M	A30	A30		7	A	1	B	A30	Wound	Wound	whole	0,94603622	0,787411809	0,70695281	0,764845205	0,066817828
11	1	C	M	A15	A15		7	A	1	C	A15	LD	MeanL	Blank	0,835217953	0,777258933	0,863761246	0,763313468	0,068055324

12	1	C	M	A15	A15	7	A	1	C	A15	LP	Blank	Blank	0,92950505	0,813889921	0,743201375	0,791916646	0,057017046
13	1	C	M	A15	A15	7	A	1	C	A15	RD	MeanR	Blank	0,844603121	0,764321089	0,836299658	0,798312756	0,061265437
14	1	C	M	A15	A15	7	A	1	C	A15	RP	Blank	Blank	0,72667706	0,692781925	0,745365202	0,814307718	0,055466551
15	1	C	M	A15	A15	7	A	1	C	A15	Wound	Wound	whole	0,965206385	0,814570487	0,639686346	0,79966561	0,055486351
16	1	D	M	C	c_A45	7	C	1	D	C	LD	MeanL	Blank	0,809127092	0,754694283	0,851016223	0,719900742	0,073184778
17	1	D	M	C	c_A45	7	C	1	D	C	LP	Blank	Blank	0,84109509	0,731452823	0,881823421	0,730001331	0,07709051
18	1	D	M	C	c_A45	7	C	1	D	C	RD	MeanR	Blank	0,758146167	0,732554495	0,665899634	0,720505428	0,077177047
19	1	D	M	C	c_A45	7	C	1	D	C	RP	Blank	Blank	0,777420044	0,730110645	0,590066671	0,683978878	0,085486448
20	1	D	M	C	c_A45	7	C	1	D	C	Wound	Wound	whole	0,972516239	0,75870502	0,691889882	0,730322741	0,063523246
21	1	E	M	C	c_A30	7	C	1	E	C	LD	MeanL	Blank	0,872415423	0,79495436	0,864617169	0,725714196	0,079021604
22	1	E	M	C	c_A30	7	C	1	E	C	LP	Blank	Blank	0,923408508	0,809151351	0,895771384	0,73847501	0,075620286
23	1	E	M	C	c_A30	7	C	1	E	C	RD	MeanR	Blank	0,901049316	0,824260175	0,837720096	0,745555752	0,071723766
24	1	E	M	C	c_A30	7	C	1	E	C	RP	Blank	Blank	0,745293558	0,694756329	0,892613351	0,735545818	0,074047369
25	1	E	M	C	c_A30	7	C	1	E	C	Wound	Wound	whole	0,967523575	0,786870003	0,767567873	0,743529675	0,068768075
26	1	F	M	C	c_A15	7	C	1	F	C	LD	MeanL	Blank	0,670356095	0,634607852	0,857142031	0,759879442	0,080206758
27	1	F	M	C	c_A15	7	C	1	F	C	LP	Blank	Blank	0,955744147	0,818646491	0,883540511	0,757331909	0,074022971
28	1	F	M	C	c_A15	7	C	1	F	C	RD	MeanR	Blank	0,82478857	0,775486708	0,727587342	0,765476153	0,06194249
29	1	F	M	C	c_A15	7	C	1	F	C	RP	Blank	Blank	0,897239089	0,788581133	0,826194406	0,751437488	0,076496921
30	1	F	M	C	c_A15	7	C	1	F	C	Wound	Wound	whole	0,906890452	0,73759371	0,695360065	0,766080307	0,066547634
31	2	A	M	A45	A45	3	A	2	A	A45	LD	MeanL	Blank	0,903046668	0,800409913	0,76674813	0,74353242	0,07069497
32	2	A	M	A45	A45	3	A	2	A	A45	LP	Blank	Blank	0,981013775	0,829450428	0,755751669	0,763962556	0,064495311
33	2	A	M	A45	A45	3	A	2	A	A45	RD	MeanR	Blank	0,760067582	0,717438161	0,727208078	0,760361481	0,075409761
34	2	A	M	A45	A45	3	A	2	A	A45	RP	Blank	Blank	0,703169107	0,676904023	0,796597004	0,766100247	0,066462616
35	2	A	M	A45	A45	3	A	2	A	A45	Wound	Wound	whole	0,958642304	0,801609397	0,609859705	0,741391519	0,074825317
36	2	B	M	A30	A30	3	A	2	B	A30	LD	MeanL	Blank	0,874970019	0,814320385	0,772681832	0,710261649	0,076399078
37	2	B	M	A30	A30	3	A	2	B	A30	LP	Blank	Blank	0,834231138	0,792218387	0,750236392	0,718710404	0,091916912
38	2	B	M	A30	A30	3	A	2	B	A30	RD	MeanR	Blank	0,733870983	0,704726875	0,761868834	0,725333711	0,095993133
39	2	B	M	A30	A30	3	A	2	B	A30	RP	Blank	Blank	0,82157284	0,769532621	0,71067065	0,746553961	0,078007241

40	2	B	M	A30	A30	3	A	2	B	A30	Wound	Wound	whole	0,881211221	0,787572384	0,657092512	0,703533571	0,083152586
41	2	C	M	A15	A15	3	A	2	C	A15	LD	MeanL	Blank	0,85113579	0,774912775	0,766739726	0,749714775	0,073604112
42	2	C	M	A15	A15	3	A	2	C	A15	LP	Blank	Blank	0,95329833	0,805527449	0,730513453	0,735604234	0,08057236
43	2	C	M	A15	A15	3	A	2	C	A15	RD	MeanR	Blank	0,813398659	0,754694521	0,775852799	0,770139444	0,075451405
44	2	C	M	A15	A15	3	A	2	C	A15	RP	Blank	Blank	0,783617735	0,74790889	0,762992442	0,754116309	0,083085967
45	2	C	M	A15	A15	3	A	2	C	A15	Wound	Wound	whole	0,942857742	0,744614065	0,600493491	0,743063258	0,077553707
46	2	D	M	C	c_A45	3	C	2	D	C	LD	MeanL	Blank	0,913020313	0,830309987	0,773960233	0,754032028	0,061724972
47	2	D	M	C	c_A45	3	C	2	D	C	LP	Blank	Blank	0,819688022	0,758144081	0,758133411	0,743782747	0,073279146
48	2	D	M	C	c_A45	3	C	2	D	C	RD	MeanR	Blank	0,933651626	0,811863482	0,702544034	0,781264416	0,063865433
49	2	D	M	C	c_A45	3	C	2	D	C	RP	Blank	Blank	0,969970703	0,846432328	0,723646462	0,746207728	0,064099334
50	2	D	M	C	c_A45	3	C	2	D	C	Wound	Wound	whole	0,952218413	0,806549847	0,686698437	0,743589742	0,074267532
51	2	E	M	C	c_A30	3	C	2	E	C	LD	MeanL	Blank	0,949241579	0,831384957	0,752830088	0,74814563	0,067496933
52	2	E	M	C	c_A30	3	C	2	E	C	LP	Blank	Blank	0,906311631	0,802963197	0,754919529	0,737497987	0,072323756
53	2	E	M	C	c_A30	3	C	2	E	C	RD	MeanR	Blank	0,941998899	0,841910839	0,821206808	0,697152631	0,071225578
54	2	E	M	C	c_A30	3	C	2	E	C	RP	Blank	Blank	0,937188864	0,850294054	0,656981826	0,73536015	0,078031847
55	2	E	M	C	c_A30	3	C	2	E	C	Wound	Wound	whole	0,957799017	0,819666088	0,661338866	0,729576607	0,076533049
56	2	F	M	C	c_A15	3	C	2	F	C	LD	MeanL	Blank	0,864003062	0,794773519	0,702794313	0,783971215	0,067952569
57	2	F	M	C	c_A15	3	C	2	F	C	LP	Blank	Blank	0,945482433	0,827456534	0,706696808	0,784999376	0,070694928
58	2	F	M	C	c_A15	3	C	2	F	C	RD	MeanR	Blank	0,947308362	0,813921869	0,773669839	0,75461842	0,068262808
59	2	F	M	C	c_A15	3	C	2	F	C	RP	Blank	Blank	0,883661389	0,819139779	0,717067897	0,750928866	0,084756228
60	2	F	M	C	c_A15	3	C	2	F	C	Wound	Wound	whole	0,924203277	0,775874436	0,536707461	0,778102237	0,064748359
91	4	A	M	C	c_A45	3	C	4	A	C	LD	MeanL	Blank	0,844000161	0,778687954	0,836908579	0,73993267	0,080133628
92	4	A	M	C	c_A45	3	C	4	A	C	LP	Blank	Blank	0,803908885	0,7529338	0,812572956	0,727278024	0,078377214
93	4	A	M	C	c_A45	3	C	4	A	C	RD	MeanR	Blank	0,9438712	0,874264359	0,658774614	0,746806557	0,069968708
94	4	A	M	C	c_A45	3	C	4	A	C	RP	Blank	Blank	0,838003397	0,802341282	0,747463465	0,730074662	0,08185809
95	4	A	M	C	c_A45	3	C	4	A	C	Wound	Wound	whole	0,899941325	0,81647861	0,643429518	0,731199056	0,074111041
96	4	B	M	C	c_A30	3	C	4	B	C	LD	MeanL	Blank	0,768374085	0,742048204	0,745506942	0,704679086	0,084821065
97	4	B	M	C	c_A30	3	C	4	B	C	LP	Blank	Blank	0,825025618	0,780583918	0,784211457	0,703644504	0,071174898

98	4	B	M	C	c_A30	3	C	4	B	C	RD	MeanR	Blank	0,799410224	0,761882484	0,834263921	0,707041513	0,086118396
99	4	B	M	C	c_A30	3	C	4	B	C	RP	Blank	Blank	0,855419755	0,804319203	0,825424612	0,691490651	0,086195047
100	4	B	M	C	c_A30	3	C	4	B	C	Wound	Wound	whole	0,909242272	0,797360122	0,688788891	0,70398489	0,081526996
101	4	C	M	C	c_A15	3	C	4	C	C	LD	MeanL	Blank	0,89903295	0,818599164	0,87565285	0,717906911	0,088373865
102	4	C	M	C	c_A15	3	C	4	C	C	LP	Blank	Blank	0,910422146	0,835842013	0,918601215	0,714418075	0,077294521
103	4	C	M	C	c_A15	3	C	4	C	C	RD	MeanR	Blank	0,961292684	0,779989779	0,793720305	0,720259334	0,089920619
104	4	C	M	C	c_A15	3	C	4	C	C	RP	Blank	Blank	0,969285667	0,837037206	0,88624537	0,751036742	0,076663717
105	4	C	M	C	c_A15	3	C	4	C	C	Wound	Wound	whole	0,977865338	0,815980971	0,726335287	0,709070441	0,081003487
106	4	D	M	A45	A45	3	A	4	D	A45	LD	MeanL	Blank	0,808653355	0,78850776	0,760597169	0,692645766	0,076471379
107	4	D	M	A45	A45	3	A	4	D	A45	LP	Blank	Blank	0,775435925	0,73664099	0,801686645	0,698203164	0,080570854
108	4	D	M	A45	A45	3	A	4	D	A45	RD	MeanR	Blank	0,922286868	0,837456703	0,807908654	0,718121504	0,078067243
109	4	D	M	A45	A45	3	A	4	D	A45	RP	Blank	Blank	0,960339725	0,858586848	0,75648284	0,708420855	0,088172455
110	4	D	M	A45	A45	3	A	4	D	A45	Wound	Wound	whole	0,959369957	0,815650821	0,566567123	0,710425014	0,085244935
111	4	E	M	A30	A30	3	A	4	E	A30	LD	MeanL	Blank	0,736365199	0,69773531	0,767575383	0,726076418	0,055635935
112	4	E	M	A30	A30	3	A	4	E	A30	LP	Blank	Blank	0,905531943	0,789827526	0,850683689	0,700442837	0,065244485
113	4	E	M	A30	A30	3	A	4	E	A30	RD	MeanR	Blank	0,875082254	0,836690962	0,660793781	0,667290544	0,093792713
114	4	E	M	A30	A30	3	A	4	E	A30	RP	Blank	Blank	0,811385334	0,755629599	0,846781611	0,68957388	0,081960326
115	4	E	M	A30	A30	3	A	4	E	A30	Wound	Wound	whole	0,951333821	0,814823866	0,672468603	0,689541516	0,085536171
116	4	F	M	A15	A15	3	A	4	F	A15	LD	MeanL	Blank	0,963510156	0,844677091	0,829962254	0,737682611	0,076819994
117	4	F	M	A15	A15	3	A	4	F	A15	LP	Blank	Blank	0,65585947	0,627955854	0,831825614	0,730678063	0,073457812
118	4	F	M	A15	A15	3	A	4	F	A15	RD	MeanR	Blank	0,892076015	0,768715501	0,775658727	0,698190038	0,089578787
119	4	F	M	A15	A15	3	A	4	F	A15	RP	Blank	Blank	0,727087379	0,696614385	0,827224553	0,710162197	0,081806389
120	4	F	M	A15	A15	3	A	4	F	A15	Wound	Wound	whole	0,953246832	0,802938223	0,594509482	0,713776803	0,085371707
121	5	A	M	C	c_A45	60	C	5	A	C	LD	MeanL	Blank	0,864417791	0,770579994	0,638173699	0,756083765	0,073821269
122	5	A	M	C	c_A45	60	C	5	A	C	LP	Blank	Blank	0,844049096	0,783159375	0,804583132	0,744136826	0,077247055
123	5	A	M	C	c_A45	60	C	5	A	C	RD	MeanR	Blank	0,880584598	0,789892852	0,81508559	0,752021596	0,073889239
124	5	A	M	C	c_A45	60	C	5	A	C	RP	Blank	Blank	0,886858106	0,821256697	0,722967327	0,747520518	0,083266212
125	5	A	M	C	c_A45	60	C	5	A	C	Wound	Wound	whole	0,838613927	0,757781029	0,772240818	0,769079131	0,059444643

126	5	B	M	C	c_A15	60	C	5	B	C	LD	MeanL	Blank	0,912268281	0,836653352	0,794494748	0,744062865	0,069757179
127	5	B	M	C	c_A15	60	C	5	B	C	LP	Blank	Blank	0,896166146	0,807860613	0,793842971	0,743277845	0,075250572
128	5	B	M	C	c_A15	60	C	5	B	C	RD	MeanR	Blank	0,737371862	0,683794379	0,814429164	0,740201413	0,081061972
129	5	B	M	C	c_A15	60	C	5	B	C	RP	Blank	Blank	0,781240463	0,728189766	0,708165288	0,762432679	0,082822256
130	5	B	M	C	c_A15	60	C	5	B	C	Wound	Wound	whole	0,861045718	0,744889677	0,84347409	0,758480187	0,061383099
131	5	C	M	C	c_A30	60	C	5	C	C	LD	MeanL	Blank	0,782859385	0,730986714	0,642067432	0,780630509	0,077064367
132	5	C	M	C	c_A30	60	C	5	C	C	LP	Blank	Blank	0,951469004	0,806987464	0,71947664	0,77232963	0,069808188
133	5	C	M	C	c_A30	60	C	5	C	C	RD	MeanR	Blank	0,905774593	0,788205147	0,72936219	0,783684626	0,07136775
134	5	C	M	C	c_A30	60	C	5	C	C	RP	Blank	Blank	0,838852167	0,766751647	0,671166122	0,772070731	0,080728952
135	5	C	M	C	c_A30	60	C	5	C	C	Wound	Wound	whole	0,922906041	0,833558857	0,704206586	0,809364872	0,044360167
136	5	D	M	A45	A45	60	A	5	D	A45	LD	MeanL	Blank	0,913238049	0,7883237	0,649091601	0,774631036	0,080445697
137	5	D	M	A45	A45	60	A	5	D	A45	LP	Blank	Blank	0,800937593	0,75312525	0,603245139	0,769445144	0,071967393
138	5	D	M	A45	A45	60	A	5	D	A45	RD	MeanR	Blank	0,771317184	0,722686231	0,616677701	0,755035587	0,078515698
139	5	D	M	A45	A45	60	A	5	D	A45	RP	Blank	Blank	0,825739682	0,780903578	0,623921931	0,75266053	0,084134934
140	5	D	M	A45	A45	60	A	5	D	A45	Wound	Wound	whole	0,876834989	0,764473915	0,560580611	0,786809816	0,057136351
141	5	E	M	A15	A15	60	A	5	E	A15	LD	MeanL	Blank	0,796616137	0,719049931	0,726538956	0,765209399	0,076201923
142	5	E	M	A15	A15	60	A	5	E	A15	LP	Blank	Blank	0,906829774	0,783068299	0,740677714	0,770040339	0,064398868
143	5	E	M	A15	A15	60	A	5	E	A15	RD	MeanR	Blank	0,969059527	0,794436872	0,755535126	0,737749913	0,088207375
144	5	E	M	A15	A15	60	A	5	E	A15	RP	Blank	Blank	0,848690391	0,778431296	0,735985398	0,728206354	0,092846781
145	5	E	M	A15	A15	60	A	5	E	A15	Wound	Wound	whole	0,861657321	0,780875623	0,658312857	0,784296632	0,050827425
146	5	F	M	A30	A30	60	A	5	F	A30	LD	MeanL	Blank	0,856218934	0,78894645	0,698021829	0,775019634	0,079920412
147	5	F	M	A30	A30	60	A	5	F	A30	LP	Blank	Blank	0,937362611	0,781424165	0,775132179	0,782625907	0,064396686
148	5	F	M	A30	A30	60	A	5	F	A30	RD	MeanR	Blank	0,889288783	0,795570493	0,740567505	0,767406902	0,077286024
149	5	F	M	A30	A30	60	A	5	F	A30	RP	Blank	Blank	0,814393163	0,757685125	0,668982327	0,755655201	0,089593581
150	5	F	M	A30	A30	60	A	5	F	A30	Wound	Wound	whole	0,876070797	0,794756651	0,789165676	0,797666229	0,047510694
181	7	A	M	C	c_A15	3	C	7	A	C	LD	MeanL	Blank	0,652589083	0,641493797	0,648032606	0,74305054	0,058325776
182	7	A	M	C	c_A15	3	C	7	A	C	LP	Blank	Blank	0,723785281	0,699163675	0,731151164	0,728024572	0,07391833
183	7	A	M	C	c_A15	3	C	7	A	C	RD	MeanR	Blank	0,728672445	0,663903058	0,697527826	0,74821666	0,071892321

184	7	A	M	C	c_A15	3	C	7	A	C	RP	Blank	Blank	0,788456202	0,757982075	0,68245846	0,747049597	0,069716124
185	7	A	M	C	c_A15	3	C	7	A	C	Wound	Wound	whole	0,848897338	0,764091313	0,610177636	0,745109353	0,067993923
186	7	B	M	C	c_A30	3	C	7	B	C	LD	MeanL	Blank	0,880590558	0,806458175	0,801780999	0,65641645	0,101296447
187	7	B	M	C	c_A30	3	C	7	B	C	LP	Blank	Blank	0,829407454	0,790678144	0,826432884	0,684931791	0,091003337
188	7	B	M	C	c_A30	3	C	7	B	C	RD	MeanR	Blank	0,800540268	0,756972194	0,871911466	0,715446457	0,077940016
189	7	B	M	C	c_A30	3	C	7	B	C	RP	Blank	Blank	0,857866287	0,77551949	0,808540642	0,732715346	0,077546156
190	7	B	M	C	c_A30	3	C	7	B	C	Wound	Wound	whole	0,839506209	0,758424103	0,555008948	0,720973639	0,08075209
191	7	C	M	C	c_A45	3	C	7	C	C	LD	MeanL	Blank	0,784860611	0,716402709	0,765264332	0,711777843	0,091759271
192	7	C	M	C	c_A45	3	C	7	C	C	LP	Blank	Blank	0,926311195	0,815205812	0,788579702	0,69654249	0,096503328
193	7	C	M	C	c_A45	3	C	7	C	C	RD	MeanR	Blank	0,863744438	0,775673628	0,796590567	0,718104373	0,084471725
194	7	C	M	C	c_A45	3	C	7	C	C	RP	Blank	Blank	0,960582554	0,829372287	0,757805824	0,696915033	0,096577858
195	7	C	M	C	c_A45	3	C	7	C	C	Wound	Wound	whole	0,957828343	0,779709935	0,604191363	0,708262694	0,09557917
196	7	D	M	A15	A15	3	A	7	D	A15	LD	MeanL	Blank	0,906092167	0,825021863	0,782060206	0,741039652	0,078620866
197	7	D	M	A15	A15	3	A	7	D	A15	LP	Blank	Blank	0,80761683	0,736504316	0,708764136	0,73620546	0,077374531
198	7	D	M	A15	A15	3	A	7	D	A15	RD	MeanR	Blank	0,778836608	0,727102339	0,671403408	0,717583614	0,063918847
199	7	D	M	A15	A15	3	A	7	D	A15	RP	Blank	Blank	0,847904921	0,80114466	0,687094629	0,711721885	0,071014773
200	7	D	M	A15	A15	3	A	7	D	A15	Wound	Wound	whole	0,945207298	0,765484989	0,49097243	0,772923738	0,07613102
201	7	E	M	A30	A30	3	A	7	E	A30	LD	MeanL	Blank	0,862309217	0,785158455	0,855323493	0,735853542	0,071308608
202	7	E	M	A30	A30	3	A	7	E	A30	LP	Blank	Blank	0,920098007	0,799762726	0,828745842	0,725558808	0,08240052
203	7	E	M	A30	A30	3	A	7	E	A30	RD	MeanR	Blank	0,72989279	0,689832807	0,796039283	0,692859979	0,083155062
204	7	E	M	A30	A30	3	A	7	E	A30	RP	Blank	Blank	0,769358933	0,735823452	0,847964287	0,682978113	0,084402963
205	7	E	M	A30	A30	3	A	7	E	A30	Wound	Wound	whole	0,922739387	0,766004562	0,68532151	0,725487368	0,082864359
206	7	F	M	A45	A45	3	A	7	F	A45	LD	MeanL	Blank	0,642087817	0,333742142	0,000782466	0,881447964	0,120639012
207	7	F	M	A45	A45	3	A	7	F	A45	LP	Blank	Blank	0,779986084	0,675683796	0,003924319	0,956848112	0,012895177
208	7	F	M	A45	A45	3	A	7	F	A45	RD	MeanR	Blank	0,690651178	0,671295345	0,777050495	0,726331238	0,08537351
209	7	F	M	A45	A45	3	A	7	F	A45	RP	Blank	Blank	0,743634999	0,723027706	0,727828979	0,725738285	0,076378182
210	7	F	M	A45	A45	3	A	7	F	A45	Wound	Wound	whole	0,846409202	0,773598969	0,815735638	0,705968205	0,083714931
301	11	A	M	A45	A45	14	A	11	A	A45	LD	MeanL	Blank	0,636250198	0,528487444	0,069848835	0,77660264	0,063688351

302	11	A	M	A45	A45	14	A	11	A	A45	LP	Blank	Blank	0,565767765	0,508108675	0,08126533	0,770300072	0,057478626
303	11	A	M	A45	A45	14	A	11	A	A45	RD	MeanR	Blank	0,564933956	0,539846778	0,345677167	0,769259861	0,038330606
304	11	A	M	A45	A45	14	A	11	A	A45	RP	Blank	Blank	0,787245929	0,727131665	0,247792125	0,757538585	0,05301177
305	11	A	M	A45	A45	14	A	11	A	A45	Wound	Wound	whole	0,732450485	0,621425927	0,26775229	0,785339941	0,048158701
306	11	B	M	A15	A15	14	A	11	B	A15	LD	MeanL	Blank	0,774920702	0,70148313	0,672148526	0,785550206	0,040622583
307	11	B	M	A15	A15	14	A	11	B	A15	LP	Blank	Blank	0,914062083	0,737968683	0,632750273	0,772920362	0,048848428
308	11	B	M	A15	A15	14	A	11	B	A15	RD	MeanR	Blank	0,882308185	0,721879661	0,692071319	0,766839591	0,051417182
309	11	B	M	A15	A15	14	A	11	B	A15	RP	Blank	Blank	0,699212313	0,67048651	0,631929755	0,770169847	0,05820092
310	11	B	M	A15	A15	14	A	11	B	A15	Wound	Wound	whole	0,854934394	0,705327392	0,466745079	0,779986034	0,046211478
311	11	C	M	A30	A30	14	A	11	C	A30	LD	MeanL	Blank	0,921494722	0,79155004	0,574169099	0,781258474	0,045306744
312	11	C	M	A30	A30	14	A	11	C	A30	LP	Blank	Blank	0,91977793	0,841782749	0,614836752	0,779020291	0,035788205
313	11	C	M	A30	A30	14	A	11	C	A30	RD	MeanR	Blank	0,869608045	0,766093373	0,679051638	0,776643879	0,043657547
314	11	C	M	A30	A30	14	A	11	C	A30	RP	Blank	Blank	0,834908664	0,736844778	0,665566742	0,761094764	0,056993614
315	11	C	M	A30	A30	14	A	11	C	A30	Wound	Wound	whole	0,841331899	0,736714482	0,369727284	0,782616427	0,035305423
316	11	D	M	C	c_A45	14	C	11	D	C	LD	MeanL	Blank	0,886967182	0,779286087	0,652813613	0,766343301	0,047563606
317	11	D	M	C	c_A45	14	C	11	D	C	LP	Blank	Blank	0,810495377	0,743405461	0,608078897	0,775372018	0,039751902
318	11	D	M	C	c_A45	14	C	11	D	C	RD	MeanR	Blank	0,823897421	0,747128606	0,583961189	0,769157213	0,035686007
319	11	D	M	C	c_A45	14	C	11	D	C	RP	Blank	Blank	0,954736531	0,788797498	0,546358705	0,759988211	0,062433783
320	11	D	M	C	c_A45	14	C	11	D	C	Wound	Wound	whole	0,959754527	0,685732126	0,41894123	0,779761397	0,032597351
321	11	E	M	C	c_A15	14	C	11	E	C	LD	MeanL	Blank	0,894592881	0,758536756	0,696995914	0,769858306	0,052918739
322	11	E	M	C	c_A15	14	C	11	E	C	LP	Blank	Blank	0,84777391	0,716682017	0,642241299	0,744062987	0,063608819
323	11	E	M	C	c_A15	14	C	11	E	C	RD	MeanR	Blank	0,834845841	0,742713392	0,680206001	0,769349117	0,047914221
324	11	E	M	C	c_A15	14	C	11	E	C	RP	Blank	Blank	0,813523054	0,761763275	0,729192793	0,775626498	0,048249398
325	11	E	M	C	c_A15	14	C	11	E	C	Wound	Wound	whole	0,856233597	0,733045876	0,538259387	0,777508293	0,041808458
326	11	F	M	C	c_A30	14	C	11	F	C	LD	MeanL	Blank	0,802111745	0,735697031	0,753682077	0,772377667	0,050894669
327	11	F	M	C	c_A30	14	C	11	F	C	LP	Blank	Blank	0,948833644	0,734059453	0,655795515	0,77789076	0,052839437
328	11	F	M	C	c_A30	14	C	11	F	C	RD	MeanR	Blank	0,94038868	0,76589483	0,415703118	0,805385768	0,041550431
329	11	F	M	C	c_A30	14	C	11	F	C	RP	Blank	Blank	0,817338407	0,771311104	0,499480516	0,797919778	0,036099935

330	11	F	M	C	c_A30	14	C	11	F	C	Wound	Wound	whole	0,955944419	0,772626042	0,386984169	0,788018183	0,041480297
361	13	A	M	A45	A45	7	A	13	A	A45	LD	MeanL	Blank	0,876843989	0,805086255	0,734191477	0,707747196	0,075202155
362	13	A	M	A45	A45	7	A	13	A	A45	LP	Blank	Blank	0,779648066	0,744198263	0,683575749	0,70743209	0,081179599
363	13	A	M	A45	A45	7	A	13	A	A45	RD	MeanR	Blank	0,798335552	0,741025746	0,734258711	0,704149671	0,069878637
364	13	A	M	A45	A45	7	A	13	A	A45	RP	Blank	Blank	0,904917419	0,852656484	0,703251958	0,69235239	0,060744423
365	13	A	M	A45	A45	7	A	13	A	A45	Wound	Wound	whole	0,77067548	0,715547919	0,564335465	0,719630021	0,067343576
366	13	B	M	A30	A30	7	A	13	B	A30	LD	MeanL	Blank	0,921407938	0,792063117	0,843918681	0,700584633	0,07294799
367	13	B	M	A30	A30	7	A	13	B	A30	LP	Blank	Blank	0,78315413	0,748464882	0,869629145	0,722298061	0,069310589
368	13	B	M	A30	A30	7	A	13	B	A30	RD	MeanR	Blank	0,905326307	0,802092373	0,85731101	0,695812975	0,0830868
369	13	B	M	A30	A30	7	A	13	B	A30	RP	Blank	Blank	0,917682707	0,821567655	0,803308666	0,705187917	0,084075755
370	13	B	M	A30	A30	7	A	13	B	A30	Wound	Wound	whole	0,950568557	0,787941039	0,746391475	0,718758034	0,067648095
371	13	C	M	A15	A15	7	A	13	C	A15	LD	MeanL	Blank	0,895747781	0,778385222	0,870949924	0,679349389	0,088810498
372	13	C	M	A15	A15	7	A	13	C	A15	LP	Blank	Blank	0,92152071	0,814180195	0,807677269	0,66626522	0,085358429
373	13	C	M	A15	A15	7	A	13	C	A15	RD	MeanR	Blank	0,918857813	0,804162383	0,854070544	0,668407569	0,087568148
374	13	C	M	A15	A15	7	A	13	C	A15	RP	Blank	Blank	0,936720014	0,83940357	0,83234489	0,700492986	0,089283531
375	13	C	M	A15	A15	7	A	13	C	A15	Wound	Wound	whole	0,954408407	0,828429401	0,714836776	0,671952521	0,073425565
376	13	D	M	C	c_A45	7	C	13	D	C	LD	MeanL	Blank	0,960546136	0,857859433	0,820493042	0,723991202	0,080248841
377	13	D	M	C	c_A45	7	C	13	D	C	LP	Blank	Blank	0,779510975	0,737042665	0,839295387	0,720915101	0,061548788
378	13	D	M	C	c_A45	7	C	13	D	C	RD	MeanR	Blank	0,805524111	0,760771871	0,748038948	0,749777649	0,067587896
379	13	D	M	C	c_A45	7	C	13	D	C	RP	Blank	Blank	0,738412857	0,704592645	0,80267626	0,713606976	0,069981309
380	13	D	M	C	c_A45	7	C	13	D	C	Wound	Wound	whole	0,842964649	0,690379858	0,610189199	0,715618727	0,070461413
381	13	E	M	C	c_A30	7	C	13	E	C	LD	MeanL	Blank	0,908676922	0,822668612	0,866842926	0,712590373	0,077537291
382	13	E	M	C	c_A30	7	C	13	E	C	LP	Blank	Blank	0,878006399	0,79274416	0,858610034	0,694587315	0,085496765
383	13	E	M	C	c_A30	7	C	13	E	C	RD	MeanR	Blank	0,924730003	0,823512912	0,823905826	0,696362446	0,071605893
384	13	E	M	C	c_A30	7	C	13	E	C	RP	Blank	Blank	0,899586678	0,801789165	0,676119447	0,678606262	0,081995764
385	13	E	M	C	c_A30	7	C	13	E	C	Wound	Wound	whole	0,925599158	0,786210775	0,760697067	0,690271569	0,073452563
386	13	F	M	C	c_A15	7	C	13	F	C	LD	MeanL	Blank	0,900516331	0,788192451	0,896575928	0,728853563	0,073763796
387	13	F	M	C	c_A15	7	C	13	F	C	LP	Blank	Blank	0,676790416	0,652627051	0,925584614	0,661512345	0,074343857

388	13	F	M	C	c_A15	7	C	13	F	C	RD	MeanR	Blank	0,878855586	0,843471766	0,672495663	0,615049933	0,097453178
389	13	F	M	C	c_A15	7	C	13	F	C	RP	Blank	Blank	0,879822493	0,773835421	0,754191697	0,643392692	0,092135579
390	13	F	M	C	c_A15	7	C	13	F	C	Wound	Wound	whole	0,82942605	0,719959259	0,600885808	0,647112152	0,071520589
391	14	A	M	A15	A15	3	A	14	A	A15	LD	MeanL	Blank	0,826712132	0,780882716	0,827030063	0,641083017	0,083654352
392	14	A	M	A15	A15	3	A	14	A	A15	LP	Blank	Blank	0,725440502	0,700632393	0,743830621	0,638863919	0,092557685
393	14	A	M	A15	A15	3	A	14	A	A15	RD	MeanR	Blank	0,893091321	0,782980084	0,855249465	0,708166514	0,076859367
394	14	A	M	A15	A15	3	A	14	A	A15	RP	Blank	Blank	0,902234316	0,796930432	0,724192142	0,696573553	0,073096518
395	14	A	M	A15	A15	3	A	14	A	A15	Wound	Wound	whole	0,922884524	0,767683268	0,696941435	0,706489412	0,078810447
396	14	B	M	A30	A30	3	A	14	B	A30	LD	MeanL	Blank	0,874988616	0,8319664	0,743449926	0,634015564	0,102123763
397	14	B	M	A30	A30	3	A	14	B	A30	LP	Blank	Blank	0,827031553	0,784732699	0,770985484	0,624101133	0,100143108
398	14	B	M	A30	A30	3	A	14	B	A30	RD	MeanR	Blank	0,960546255	0,77739346	0,851744771	0,685456089	0,077729584
399	14	B	M	A30	A30	3	A	14	B	A30	RP	Blank	Blank	0,894355178	0,808003068	0,810657561	0,690156014	0,083192513
400	14	B	M	A30	A30	3	A	14	B	A30	Wound	Wound	whole	0,921435714	0,775308788	0,693081558	0,692096407	0,089380367
401	14	C	M	A45	A45	3	A	14	C	A45	LD	MeanL	Blank	0,86790961	0,787424326	0,833921552	0,713980674	0,083058218
402	14	C	M	A45	A45	3	A	14	C	A45	LP	Blank	Blank	0,931425214	0,816303372	0,80727917	0,680959704	0,099720919
403	14	C	M	A45	A45	3	A	14	C	A45	RD	MeanR	Blank	0,898426414	0,815697789	0,835556984	0,685768781	0,084345216
404	14	C	M	A45	A45	3	A	14	C	A45	RP	Blank	Blank	0,761614382	0,704904318	0,741983473	0,675925347	0,092481201
405	14	C	M	A45	A45	3	A	14	C	A45	Wound	Wound	whole	0,88236326	0,74915719	0,53342998	0,709608232	0,082084743
406	14	D	M	C	c_A15	3	C	14	D	C	LD	MeanL	Blank	0,834783316	0,786009967	0,746999085	0,727572534	0,078526401
407	14	D	M	C	c_A15	3	C	14	D	C	LP	Blank	Blank	0,794223785	0,725957215	0,830126107	0,720261195	0,074322119
408	14	D	M	C	c_A15	3	C	14	D	C	RD	MeanR	Blank	0,837527096	0,773861289	0,803743958	0,708535039	0,07783091
409	14	D	M	C	c_A15	3	C	14	D	C	RP	Blank	Blank	0,88142395	0,826347172	0,810444117	0,674158827	0,085081136
410	14	D	M	C	c_A15	3	C	14	D	C	Wound	Wound	whole	0,90329057	0,751083791	0,68985945	0,719339877	0,074199772
411	14	E	M	C	c_A30	3	C	14	E	C	LD	MeanL	Blank	0,8994506	0,82092911	0,793309629	0,75132516	0,067521424
412	14	E	M	C	c_A30	3	C	14	E	C	LP	Blank	Blank	0,799309134	0,749612451	0,826845646	0,724346314	0,084133436
413	14	E	M	C	c_A30	3	C	14	E	C	RD	MeanR	Blank	0,871305346	0,799012661	0,864976227	0,684552247	0,078385293
414	14	E	M	C	c_A30	3	C	14	E	C	RP	Blank	Blank	0,949617386	0,812535405	0,835365534	0,67418951	0,095895143
415	14	E	M	C	c_A30	3	C	14	E	C	Wound	Wound	whole	0,899609864	0,758539855	0,716856182	0,716075814	0,081603278

416	14	F	M	C	c_A45	3	C	14	F	C	LD	MeanL	Blank	0,933932245	0,802845895	0,872079492	0,715386296	0,087383439
417	14	F	M	C	c_A45	3	C	14	F	C	LP	Blank	Blank	0,923721313	0,822626293	0,847814977	0,696739698	0,092656144
418	14	F	M	C	c_A45	3	C	14	F	C	RD	MeanR	Blank	0,801893294	0,748629332	0,834736288	0,697495247	0,089100594
419	14	F	M	C	c_A45	3	C	14	F	C	RP	Blank	Blank	0,961747646	0,803231776	0,801340878	0,670234078	0,104303907
420	14	F	M	C	c_A45	3	C	14	F	C	Wound	Wound	whole	0,97934413	0,79075247	0,599412024	0,697752306	0,090480298
421	15	A	M	A30	A30	14	A	15	A	A30	LD	MeanL	Blank	0,925734282	0,801921427	0,783916593	0,730907646	0,067170595
422	15	A	M	A30	A30	14	A	15	A	A30	LP	Blank	Blank	0,915890336	0,813270032	0,796236038	0,742158271	0,061041209
423	15	A	M	A30	A30	14	A	15	A	A30	RD	MeanR	Blank	0,750914097	0,704457045	0,580069184	0,731454016	0,087783703
424	15	A	M	A30	A30	14	A	15	A	A30	RP	Blank	Blank	0,854869187	0,767637312	0,549498379	0,745675875	0,078609449
425	15	A	M	A30	A30	14	A	15	A	A30	Wound	Wound	whole	0,944461524	0,78534168	0,743130624	0,751388364	0,065489716
426	15	B	M	A45	A45	14	A	15	B	A45	LD	MeanL	Blank	0,815691531	0,751942694	0,810985923	0,725711829	0,054395495
427	15	B	M	A45	A45	14	A	15	B	A45	LP	Blank	Blank	0,858586133	0,762901068	0,837732494	0,729249241	0,07055751
428	15	B	M	A45	A45	14	A	15	B	A45	RD	MeanR	Blank	0,761308908	0,686254323	0,852129221	0,731720116	0,061383222
429	15	B	M	A45	A45	14	A	15	B	A45	RP	Blank	Blank	0,792760015	0,718800128	0,707081318	0,726025459	0,084557164
430	15	B	M	A45	A45	14	A	15	B	A45	Wound	Wound	whole	0,900123	0,768297851	0,793781519	0,724435071	0,046838468
431	15	C	M	A15	A15	14	A	15	C	A15	LD	MeanL	Blank	0,923198938	0,823394716	0,838434935	0,723360517	0,072154492
432	15	C	M	A15	A15	14	A	15	C	A15	LP	Blank	Blank	0,954596102	0,791530788	0,845891297	0,72840434	0,052607151
433	15	C	M	A15	A15	14	A	15	C	A15	RD	MeanR	Blank	0,948966026	0,809786081	0,802199721	0,723564142	0,054150017
434	15	C	M	A15	A15	14	A	15	C	A15	RP	Blank	Blank	0,929246247	0,764637351	0,780438304	0,763226016	0,059309545
435	15	C	M	A15	A15	14	A	15	C	A15	Wound	Wound	whole	0,949623466	0,798702598	0,786068797	0,717527463	0,058088921
436	15	D	M	C	c_A30	14	C	15	D	C	LD	MeanL	Blank	0,849499583	0,776564538	0,69043237	0,71097814	0,091625425
437	15	D	M	C	c_A30	14	C	15	D	C	LP	Blank	Blank	0,765176594	0,689221263	0,59108752	0,715741827	0,082194972
438	15	D	M	C	c_A30	14	C	15	D	C	RD	MeanR	Blank	0,860526145	0,789580643	0,779523611	0,735361404	0,076559657
439	15	D	M	C	c_A30	14	C	15	D	C	RP	Blank	Blank	0,90954411	0,751221836	0,803352594	0,717891343	0,080105057
440	15	D	M	C	c_A30	14	C	15	D	C	Wound	Wound	whole	0,917694747	0,745770752	0,661227584	0,738908229	0,064775856
441	15	E	M	C	c_A45	14	C	15	E	C	LD	MeanL	Blank	0,882294953	0,792629957	0,789295912	0,691371326	0,084043714
442	15	E	M	C	c_A45	14	C	15	E	C	LP	Blank	Blank	0,916215003	0,776545107	0,795456171	0,677072583	0,097117891
443	15	E	M	C	c_A45	14	C	15	E	C	RD	MeanR	Blank	0,932714403	0,81235975	0,899528086	0,70101221	0,069584751

444	15	E	M	C	c_A45	14	C	15	E	C	RP	Blank	Blank	0,967872858	0,801986277	0,851324379	0,728148604	0,065026242
445	15	E	M	C	c_A45	14	C	15	E	C	Wound	Wound	whole	0,91638279	0,787219644	0,788048267	0,702901873	0,063654126
446	15	F	M	C	c_A15	14	C	15	F	C	LD	MeanL	Blank	0,924864948	0,778708696	0,765458465	0,751916585	0,070200366
447	15	F	M	C	c_A15	14	C	15	F	C	LP	Blank	Blank	0,959623575	0,778969109	0,720787108	0,708921138	0,090193331
448	15	F	M	C	c_A15	14	C	15	F	C	RD	MeanR	Blank	0,89841336	0,784440041	0,759784043	0,702122044	0,090124711
449	15	F	M	C	c_A15	14	C	15	F	C	RP	Blank	Blank	0,950297832	0,771817267	0,76579231	0,740111432	0,073503956
450	15	F	M	C	c_A15	14	C	15	F	C	Wound	Wound	whole	0,932767272	0,772283375	0,714589834	0,727467541	0,069983047
451	16	A	M	A15	A15	3	A	16	A	A15	LD	MeanL	Blank	0,837111473	0,762999058	0,771505654	0,720805225	0,065953715
452	16	A	M	A15	A15	3	A	16	A	A15	LP	Blank	Blank	0,799067497	0,735498309	0,779690385	0,664811924	0,073029068
453	16	A	M	A15	A15	3	A	16	A	A15	RD	MeanR	Blank	0,879863381	0,799940109	0,775469244	0,689060114	0,08755646
454	16	A	M	A15	A15	3	A	16	A	A15	RP	Blank	Blank	0,800395429	0,757538795	0,824889898	0,682858851	0,092732783
455	16	A	M	A15	A15	3	A	16	A	A15	Wound	Wound	whole	0,894986212	0,787789106	0,699692607	0,683009651	0,079086268
456	16	B	M	A30	A30	3	A	16	B	A30	LD	MeanL	Blank	0,841314435	0,802228391	0,80329442	0,665663266	0,0857161
457	16	B	M	A30	A30	3	A	16	B	A30	LP	Blank	Blank	0,778090954	0,724799812	0,703297019	0,668983629	0,081542024
458	16	B	M	A30	A30	3	A	16	B	A30	RD	MeanR	Blank	0,876363754	0,733387232	0,835653901	0,715311804	0,06587735
459	16	B	M	A30	A30	3	A	16	B	A30	RP	Blank	Blank	0,847442031	0,764138997	0,814037859	0,716346201	0,089209631
460	16	B	M	A30	A30	3	A	16	B	A30	Wound	Wound	whole	0,887140214	0,745612025	0,640269876	0,71224257	0,080771339
461	16	C	M	A45	A45	3	A	16	C	A45	LD	MeanL	Blank	0,801964641	0,740707338	0,858533263	0,722400487	0,079725974
462	16	C	M	A45	A45	3	A	16	C	A45	LP	Blank	Blank	0,922876716	0,820158839	0,809014976	0,706462452	0,0786764
463	16	C	M	A45	A45	3	A	16	C	A45	RD	MeanR	Blank	0,853586018	0,778811991	0,845803499	0,728182327	0,064941783
464	16	C	M	A45	A45	3	A	16	C	A45	RP	Blank	Blank	0,79708159	0,751903355	0,844841659	0,734751282	0,074027582
465	16	C	M	A45	A45	3	A	16	C	A45	Wound	Wound	whole	0,907536149	0,794551969	0,694524705	0,717666238	0,085327197
466	16	D	M	C	c_A15	3	C	16	D	C	LD	MeanL	Blank	0,764492571	0,71075958	0,765607476	0,731644623	0,078159809
467	16	D	M	C	c_A15	3	C	16	D	C	LP	Blank	Blank	0,896120131	0,765117168	0,856207252	0,721992297	0,074776231
468	16	D	M	C	c_A15	3	C	16	D	C	RD	MeanR	Blank	0,816933811	0,78700316	0,687818825	0,654945601	0,0829339
469	16	D	M	C	c_A15	3	C	16	D	C	RP	Blank	Blank	0,836630702	0,797023535	0,804182053	0,711185635	0,065638681
470	16	D	M	C	c_A15	3	C	16	D	C	Wound	Wound	whole	0,886785746	0,773383796	0,672815204	0,709870122	0,078162907
471	16	E	M	C	c_A30	3	C	16	E	C	LD	MeanL	Blank	0,866225123	0,765874326	0,822827041	0,737574366	0,06842461

472	16	E	M	C	c_A30	3	C	16	E	C	LP	Blank	Blank	0,902182221	0,804686785	0,846483588	0,681008105	0,086480417
473	16	E	M	C	c_A30	3	C	16	E	C	RD	MeanR	Blank	0,965351105	0,856717348	0,832538426	0,681395592	0,079010917
474	16	E	M	C	c_A30	3	C	16	E	C	RP	Blank	Blank	0,875356793	0,805599928	0,834699988	0,685444661	0,085115963
475	16	E	M	C	c_A30	3	C	16	E	C	Wound	Wound	whole	0,956129491	0,806690454	0,749597132	0,667134389	0,081932777
476	16	F	M	C	c_A45	3	C	16	F	C	LD	MeanL	Blank	0,979174733	0,820912719	0,848333538	0,751231427	0,074874881
477	16	F	M	C	c_A45	3	C	16	F	C	LP	Blank	Blank	0,862245917	0,790137768	0,861635566	0,707611706	0,083318663
478	16	F	M	C	c_A45	3	C	16	F	C	RD	MeanR	Blank	0,916359782	0,830483854	0,889343679	0,693346139	0,090259105
479	16	F	M	C	c_A45	3	C	16	F	C	RP	Blank	Blank	0,902547061	0,817374647	0,775528967	0,711325985	0,076422789
480	16	F	M	C	c_A45	3	C	16	F	C	Wound	Wound	whole	0,859700441	0,768234074	0,708252072	0,698107522	0,090679351
511	18	A	M	C	c_A45	60	C	18	A	C	LD	MeanL	Blank	0,949131727	0,811790168	0,685183227	0,747135919	0,065547702
512	18	A	M	C	c_A45	60	C	18	A	C	LP	Blank	Blank	0,924260795	0,812074423	0,649560332	0,748984805	0,060260682
513	18	A	M	C	c_A45	60	C	18	A	C	RD	MeanR	Blank	0,783618569	0,729452133	0,728161693	0,751701306	0,069249682
514	18	A	M	C	c_A45	60	C	18	A	C	RP	Blank	Blank	0,852924824	0,762213409	0,733553708	0,72447984	0,078757744
515	18	A	M	C	c_A45	60	C	18	A	C	Wound	Wound	whole	0,940880299	0,787936747	0,565981328	0,770864403	0,054092564
516	18	B	M	C	c_A15	60	C	18	B	C	LD	MeanL	Blank	0,957712173	0,814311028	0,690782845	0,737820978	0,070763142
517	18	B	M	C	c_A15	60	C	18	B	C	LP	Blank	Blank	0,907559514	0,779538035	0,722180247	0,72570494	0,066438526
518	18	B	M	C	c_A15	60	C	18	B	C	RD	MeanR	Blank	0,953915894	0,750220418	0,780925512	0,741238026	0,046748539
519	18	B	M	C	c_A15	60	C	18	B	C	RP	Blank	Blank	0,883710325	0,790563345	0,672765434	0,714937385	0,077434585
520	18	B	M	C	c_A15	60	C	18	B	C	Wound	Wound	whole	0,957374096	0,808308721	0,654959924	0,758850916	0,043166262
521	18	C	M	C	c_A30	60	C	18	C	C	LD	MeanL	Blank	0,835570335	0,76097852	0,744745731	0,735110644	0,071525189
522	18	C	M	C	c_A30	60	C	18	C	C	LP	Blank	Blank	0,719015002	0,689799309	0,799765646	0,728928141	0,064289015
523	18	C	M	C	c_A30	60	C	18	C	C	RD	MeanR	Blank	0,776587009	0,717214704	0,845837951	0,725336967	0,068769873
524	18	C	M	C	c_A30	60	C	18	C	C	RP	Blank	Blank	0,718647718	0,685202539	0,793366909	0,698040614	0,085282455
525	18	C	M	C	c_A30	60	C	18	C	C	Wound	Wound	whole	0,80491519	0,709191799	0,61347419	0,754745867	0,053952534
526	18	D	M	A45	A45	60	A	18	D	A45	LD	MeanL	Blank	0,984510422	0,851068676	0,754078686	0,749343996	0,062396803
527	18	D	M	A45	A45	60	A	18	D	A45	LP	Blank	Blank	0,866337001	0,786649168	0,719467402	0,756182708	0,06370851
528	18	D	M	A45	A45	60	A	18	D	A45	RD	MeanR	Blank	0,911955059	0,782446563	0,729134738	0,746048543	0,06199412
529	18	D	M	A45	A45	60	A	18	D	A45	RP	Blank	Blank	0,841611743	0,777141213	0,595739782	0,754740652	0,058257427

530	18	D	M	A45	A45	60	A	18	D	A45	Wound	Wound	whole	0,894731343	0,778181851	0,694913566	0,762972609	0,053234298
531	18	E	M	A15	A15	60	A	18	E	A15	LD	MeanL	Blank	0,834830403	0,771133959	0,739580095	0,728099321	0,070117128
532	18	E	M	A15	A15	60	A	18	E	A15	LP	Blank	Blank	0,860275209	0,765479505	0,737410307	0,734826416	0,070052763
533	18	E	M	A15	A15	60	A	18	E	A15	RD	MeanR	Blank	0,904718697	0,810806453	0,789448082	0,748355829	0,0603665
534	18	E	M	A15	A15	60	A	18	E	A15	RP	Blank	Blank	0,844720483	0,793422282	0,727111042	0,737581005	0,068447658
535	18	E	M	A15	A15	60	A	18	E	A15	Wound	Wound	whole	0,877327383	0,792827606	0,724416375	0,749671977	0,049871551
536	18	F	M	A30	A30	60	A	18	F	A30	LD	MeanL	Blank	0,763410807	0,716375709	0,733378351	0,73182436	0,071935743
537	18	F	M	A30	A30	60	A	18	F	A30	LP	Blank	Blank	0,805869758	0,709085703	0,724216938	0,734761092	0,068201611
538	18	F	M	A30	A30	60	A	18	F	A30	RD	MeanR	Blank	0,708224475	0,6714046	0,828247011	0,723909522	0,058537567
539	18	F	M	A30	A30	60	A	18	F	A30	RP	Blank	Blank	0,98728466	0,788013518	0,768754423	0,702034609	0,065219768
540	18	F	M	A30	A30	60	A	18	F	A30	Wound	Wound	whole	0,869056582	0,797251105	0,650659978	0,767382788	0,043495479
571	20	A	M	A15	A15	14	A	20	A	A15	LD	MeanL	Blank	0,934400797	0,838766098	0,854683638	0,758109945	0,064312443
572	20	A	M	A15	A15	14	A	20	A	A15	LP	Blank	Blank	0,949282229	0,825398326	0,802272737	0,75770801	0,055030415
573	20	A	M	A15	A15	14	A	20	A	A15	RD	MeanR	Blank	0,839583933	0,73515135	0,67956388	0,741505357	0,078905143
574	20	A	M	A15	A15	14	A	20	A	A15	RP	Blank	Blank	0,817221761	0,734803796	0,642567813	0,741420333	0,074672887
575	20	A	M	A15	A15	14	A	20	A	A15	Wound	Wound	whole	0,96922344	0,78660804	0,719345093	0,771508547	0,051230889
576	20	B	M	A45	A45	14	A	20	B	A45	LD	MeanL	Blank	0,983049572	0,801605821	0,843953013	0,777508457	0,056915626
577	20	B	M	A45	A45	14	A	20	B	A45	LP	Blank	Blank	0,920814633	0,78865391	0,874325454	0,750922371	0,065853186
578	20	B	M	A45	A45	14	A	20	B	A45	RD	MeanR	Blank	0,7858603	0,722593546	0,794457555	0,737748249	0,076854782
579	20	B	M	A45	A45	14	A	20	B	A45	RP	Blank	Blank	0,881550133	0,767762184	0,789316893	0,763481874	0,059842818
580	20	B	M	A45	A45	14	A	20	B	A45	Wound	Wound	whole	0,899291992	0,708463013	0,692074656	0,764934904	0,060317233
581	20	C	M	A30	A30	14	A	20	C	A30	LD	MeanL	Blank	0,878382802	0,760569632	0,867180645	0,78366266	0,064763897
582	20	C	M	A30	A30	14	A	20	C	A30	LP	Blank	Blank	0,870763302	0,7729128	0,838281453	0,750307243	0,062511169
583	20	C	M	A30	A30	14	A	20	C	A30	RD	MeanR	Blank	0,91986239	0,805473447	0,844676971	0,753766862	0,062345226
584	20	C	M	A30	A30	14	A	20	C	A30	RP	Blank	Blank	0,962713361	0,777460277	0,828620017	0,769671472	0,057361901
585	20	C	M	A30	A30	14	A	20	C	A30	Wound	Wound	whole	0,864129782	0,73397541	0,693062425	0,775645516	0,054274957
586	20	D	M	C	c_A15	14	C	20	D	C	LD	MeanL	Blank	0,777565002	0,739974916	0,757670224	0,752295793	0,073973737
587	20	D	M	C	c_A15	14	C	20	D	C	LP	Blank	Blank	0,958320558	0,810230851	0,877546251	0,770273073	0,055828833

588	20	D	M	C	c_A15	14	C	20	D	C	RD	MeanR	Blank	0,875563741	0,816710114	0,7461797	0,743303501	0,06454701
589	20	D	M	C	c_A15	14	C	20	D	C	RP	Blank	Blank	0,81424588	0,739322305	0,703664243	0,722632874	0,080792701
590	20	D	M	C	c_A15	14	C	20	D	C	Wound	Wound	whole	0,966926992	0,767192125	0,634380519	0,780047295	0,057227882
591	20	E	M	C	c_A45	14	C	20	E	C	LD	MeanL	Blank	0,794727325	0,726619244	0,662478328	0,710301274	0,08943624
592	20	E	M	C	c_A45	14	C	20	E	C	LP	Blank	Blank	0,824136615	0,74140811	0,677415133	0,71549155	0,090334363
593	20	E	M	C	c_A45	14	C	20	E	C	RD	MeanR	Blank	0,849792242	0,778756738	0,796118021	0,758569538	0,066654583
594	20	E	M	C	c_A45	14	C	20	E	C	RP	Blank	Blank	0,838830352	0,772791624	0,751310468	0,763327066	0,072697126
595	20	E	M	C	c_A45	14	C	20	E	C	Wound	Wound	whole	0,882642329	0,70111227	0,551868021	0,773863731	0,058221568
596	20	F	M	C	c_A30	14	C	20	F	C	LD	MeanL	Blank	0,875115275	0,79104811	0,750107288	0,754911392	0,074985478
597	20	F	M	C	c_A30	14	C	20	F	C	LP	Blank	Blank	0,934195638	0,827753127	0,866042256	0,79272104	0,049524615
598	20	F	M	C	c_A30	14	C	20	F	C	RD	MeanR	Blank	0,972181976	0,829219997	0,790262043	0,768196854	0,069018816
599	20	F	M	C	c_A30	14	C	20	F	C	RP	Blank	Blank	0,931003928	0,815136492	0,809124231	0,794735083	0,059883555
600	20	F	M	C	c_A30	14	C	20	F	C	Wound	Wound	whole	0,888967633	0,751113355	0,675301135	0,794501089	0,051648521
631	22	A	M	C	c_A15	60	C	22	A	C	LD	MeanL	Blank	0,887592614	0,8418836	0,61265105	0,71712235	0,073153775
632	22	A	M	C	c_A15	60	C	22	A	C	LP	Blank	Blank	0,944425046	0,81416893	0,660503924	0,713676359	0,074576595
633	22	A	M	C	c_A15	60	C	22	A	C	RD	MeanR	Blank	0,928244531	0,840359688	0,718508661	0,697127358	0,086368935
634	22	A	M	C	c_A15	60	C	22	A	C	RP	Blank	Blank	0,798032045	0,745278955	0,708477378	0,712821576	0,078083353
635	22	A	M	C	c_A15	60	C	22	A	C	Wound	Wound	whole	0,895399868	0,769001663	0,622181356	0,720743053	0,066773923
636	22	B	M	C	c_A45	60	C	22	B	C	LD	MeanL	Blank	0,90779084	0,808360398	0,677075386	0,696228914	0,086734035
637	22	B	M	C	c_A45	60	C	22	B	C	LP	Blank	Blank	0,922973514	0,774005949	0,725339115	0,703858211	0,082828775
638	22	B	M	C	c_A45	60	C	22	B	C	RD	MeanR	Blank	0,874167979	0,747887492	0,734969616	0,71692996	0,086266814
639	22	B	M	C	c_A45	60	C	22	B	C	RP	Blank	Blank	0,869374871	0,742753506	0,699875176	0,744986241	0,062435455
640	22	B	M	C	c_A45	60	C	22	B	C	Wound	Wound	whole	0,848825216	0,761725843	0,675727785	0,751399774	0,062652566
641	22	C	M	C	c_A30	60	C	22	C	C	LD	MeanL	Blank	0,885703743	0,776510894	0,623843431	0,731268572	0,075647364
642	22	C	M	C	c_A30	60	C	22	C	C	LP	Blank	Blank	0,939528286	0,807115972	0,691729426	0,725362271	0,082635725
643	22	C	M	C	c_A30	60	C	22	C	C	RD	MeanR	Blank	0,810576439	0,735862195	0,724713564	0,73155731	0,072441189
644	22	C	M	C	c_A30	60	C	22	C	C	RP	Blank	Blank	0,866630793	0,80601114	0,724905193	0,726947705	0,083284318
645	22	C	M	C	c_A30	60	C	22	C	C	Wound	Wound	whole	0,915664315	0,779720366	0,623124719	0,745152638	0,066609077

646	22	D	M	A15	A15	60	A	22	D	A15	LD	MeanL	Blank	0,851574957	0,800001383	0,677506983	0,717317769	0,08676382
647	22	D	M	A15	A15	60	A	22	D	A15	LP	Blank	Blank	0,962107956	0,84539479	0,611865461	0,712290079	0,088565706
648	22	D	M	A15	A15	60	A	22	D	A15	RD	MeanR	Blank	0,88510704	0,795059264	0,601384997	0,699804342	0,086620799
649	22	D	M	A15	A15	60	A	22	D	A15	RP	Blank	Blank	0,936032653	0,853052735	0,629875124	0,715544423	0,088549844
650	22	D	M	A15	A15	60	A	22	D	A15	Wound	Wound	whole	0,946331084	0,813348174	0,657369673	0,708422563	0,079729303
651	22	E	M	A45	A45	60	A	22	E	A45	LD	MeanL	Blank	0,767345786	0,727533042	0,668410599	0,728392239	0,0821854
652	22	E	M	A45	A45	60	A	22	E	A45	LP	Blank	Blank	0,881638288	0,800820768	0,689690471	0,724107503	0,081256296
653	22	E	M	A45	A45	60	A	22	E	A45	RD	MeanR	Blank	0,923553824	0,795222938	0,702556372	0,733505979	0,077844864
654	22	E	M	A45	A45	60	A	22	E	A45	RP	Blank	Blank	0,720331252	0,684357226	0,650080562	0,717017631	0,088008837
655	22	E	M	A45	A45	60	A	22	E	A45	Wound	Wound	whole	0,946651578	0,795856178	0,733732581	0,735873255	0,073288547
656	22	F	M	A30	A30	60	A	22	F	A30	LD	MeanL	Blank	0,854662776	0,783579767	0,70915544	0,737613621	0,075601043
657	22	F	M	A30	A30	60	A	22	F	A30	LP	Blank	Blank	0,936963558	0,823347509	0,791545331	0,730388329	0,079881313
658	22	F	M	A30	A30	60	A	22	F	A30	RD	MeanR	Blank	0,927866042	0,812905908	0,84039259	0,730782748	0,076211989
659	22	F	M	A30	A30	60	A	22	F	A30	RP	Blank	Blank	0,768021166	0,73170644	0,672758996	0,722803853	0,079588309
660	22	F	M	A30	A30	60	A	22	F	A30	Wound	Wound	whole	0,90221411	0,8048594	0,76437515	0,751657439	0,057459424
691	24	A	M	C	c_A45	3	C	24	A	C	LD	MeanL	Blank	0,809915364	0,777314425	0,752978146	0,645610181	0,0925285
692	24	A	M	C	c_A45	3	C	24	A	C	LP	Blank	Blank	0,64485538	0,628695786	0,687552392	0,657376013	0,086106095
693	24	A	M	C	c_A45	3	C	24	A	C	RD	MeanR	Blank	0,84787643	0,774305999	0,755423903	0,663727502	0,085459069
694	24	A	M	C	c_A45	3	C	24	A	C	RP	Blank	Blank	0,840095043	0,799227357	0,618652642	0,660166635	0,096830381
695	24	A	M	C	c_A45	3	C	24	A	C	Wound	Wound	whole	0,876174629	0,759045303	0,627888858	0,656994065	0,088381857
696	24	B	M	C	c_A30	3	C	24	B	C	LD	MeanL	Blank	0,739662409	0,710890114	0,813040495	0,698368318	0,085921639
697	24	B	M	C	c_A30	3	C	24	B	C	LP	Blank	Blank	0,800256729	0,774033368	0,811795354	0,687414358	0,097130695
698	24	B	M	C	c_A30	3	C	24	B	C	RD	MeanR	Blank	0,850344181	0,792176247	0,840271592	0,701516901	0,093070466
699	24	B	M	C	c_A30	3	C	24	B	C	RP	Blank	Blank	0,811596096	0,741019905	0,821489513	0,719644193	0,080177013
700	24	B	M	C	c_A30	3	C	24	B	C	Wound	Wound	whole	0,858307958	0,750455379	0,624647677	0,708310946	0,082132773
701	24	C	M	C	c_A15	3	C	24	C	C	LD	MeanL	Blank	0,928389788	0,793462694	0,894654155	0,717126921	0,077799881
702	24	C	M	C	c_A15	3	C	24	C	C	LP	Blank	Blank	0,750732183	0,730330765	0,862714648	0,671254725	0,093899541
703	24	C	M	C	c_A15	3	C	24	C	C	RD	MeanR	Blank	0,760002077	0,725731611	0,839615762	0,717820074	0,080811862

704	24	C	M	C	c_A15	3	C	24	C	C	RP	Blank	Blank	0,821319401	0,774847448	0,877371669	0,7485651	0,06532654
705	24	C	M	C	c_A15	3	C	24	C	C	Wound	Wound	whole	0,877989292	0,729775429	0,638101995	0,699476541	0,088507827
706	24	D	M	A45	A45	3	A	24	D	A45	LD	MeanL	Blank	0,836484492	0,791080117	0,769740283	0,679466689	0,092672926
707	24	D	M	A45	A45	3	A	24	D	A45	LP	Blank	Blank	0,855887294	0,748732865	0,864805758	0,723760153	0,075870513
708	24	D	M	A45	A45	3	A	24	D	A45	RD	MeanR	Blank	0,837285399	0,812367439	0,686405003	0,702320214	0,076010956
709	24	D	M	A45	A45	3	A	24	D	A45	RP	Blank	Blank	0,89349848	0,826243579	0,753217459	0,682997717	0,072694805
710	24	D	M	A45	A45	3	A	24	D	A45	Wound	Wound	whole	0,892382562	0,763835847	0,692012072	0,739938719	0,072439552
711	24	E	M	A30	A30	3	A	24	E	A30	LD	MeanL	Blank	0,916311502	0,825876415	0,882113576	0,699010624	0,084533872
712	24	E	M	A30	A30	3	A	24	E	A30	LP	Blank	Blank	0,842195749	0,762471139	0,850243986	0,701582566	0,09197757
713	24	E	M	A30	A30	3	A	24	E	A30	RD	MeanR	Blank	0,732997417	0,703833818	0,804545999	0,664172567	0,091360599
714	24	E	M	A30	A30	3	A	24	E	A30	RP	Blank	Blank	0,821167111	0,776909411	0,747636855	0,711510566	0,08760049
715	24	E	M	A30	A30	3	A	24	E	A30	Wound	Wound	whole	0,938301563	0,79453522	0,622504413	0,688518073	0,091864163
716	24	F	M	A15	A15	3	A	24	F	A15	LD	MeanL	Blank	0,760740101	0,732464612	0,860069394	0,735289842	0,074474953
717	24	F	M	A15	A15	3	A	24	F	A15	LP	Blank	Blank	0,85785532	0,787520945	0,830373347	0,700161462	0,088298524
718	24	F	M	A15	A15	3	A	24	F	A15	RD	MeanR	Blank	0,812760293	0,785133183	0,80569607	0,712213526	0,081104235
719	24	F	M	A15	A15	3	A	24	F	A15	RP	Blank	Blank	0,923667014	0,807184041	0,838975906	0,729828801	0,081646016
720	24	F	M	A15	A15	3	A	24	F	A15	Wound	Wound	whole	0,830928385	0,764615655	0,69371593	0,706429326	0,083967904
781	27	A	M	C	c_A15	60	C	27	A	C	LD	MeanL	Blank	0,872196972	0,782275498	0,701087475	0,78207762	0,059295282
782	27	A	M	C	c_A15	60	C	27	A	C	LP	Blank	Blank	0,882730246	0,806271672	0,729740977	0,79642612	0,050677787
783	27	A	M	C	c_A15	60	C	27	A	C	RD	MeanR	Blank	0,856317163	0,796753407	0,781515121	0,787206498	0,049700319
784	27	A	M	C	c_A15	60	C	27	A	C	RP	Blank	Blank	0,802063107	0,7462551	0,645786166	0,775498592	0,061763795
785	27	A	M	C	c_A15	60	C	27	A	C	Wound	Wound	whole	0,935328007	0,752684653	0,704180658	0,797067374	0,047917361
786	27	B	M	C	c_A45	60	C	27	B	C	LD	MeanL	Blank	0,891744792	0,822683156	0,726714075	0,793328468	0,04998213
787	27	B	M	C	c_A45	60	C	27	B	C	LP	Blank	Blank	0,89134872	0,826792479	0,830822945	0,787235558	0,051822143
788	27	B	M	C	c_A45	60	C	27	B	C	RD	MeanR	Blank	0,866111517	0,80409199	0,732648432	0,790753948	0,058075808
789	27	B	M	C	c_A45	60	C	27	B	C	RP	Blank	Blank	0,91388756	0,809248924	0,736758471	0,791129449	0,055109107
790	27	B	M	C	c_A45	60	C	27	B	C	Wound	Wound	whole	0,925482213	0,757199407	0,732524991	0,791744667	0,053832834
791	27	C	M	C	c_A30	60	C	27	C	C	LD	MeanL	Blank	0,931815088	0,824445248	0,671082437	0,777104586	0,05923584

792	27	C	M	C	c_A30	60	C	27	C	C	LP	Blank	Blank	0,957564592	0,828060865	0,813989937	0,7722073	0,060901784
793	27	C	M	C	c_A30	60	C	27	C	C	RD	MeanR	Blank	0,571871996	0,526128888	0,850190341	0,765117802	0,058820136
794	27	C	M	C	c_A30	60	C	27	C	C	RP	Blank	Blank	0,773298621	0,719184279	0,768712759	0,779116679	0,057084459
795	27	C	M	C	c_A30	60	C	27	C	C	Wound	Wound	whole	0,894631922	0,806967199	0,732269049	0,805818543	0,04677815
796	27	D	M	A15	A15	60	A	27	D	A15	LD	MeanL	Blank	0,886047304	0,819470823	0,603377521	0,778746082	0,059099192
797	27	D	M	A15	A15	60	A	27	D	A15	LP	Blank	Blank	0,916038215	0,820938885	0,682971895	0,792271774	0,047911131
798	27	D	M	A15	A15	60	A	27	D	A15	RD	MeanR	Blank	0,877634883	0,817949831	0,559223533	0,776290802	0,057538549
799	27	D	M	A15	A15	60	A	27	D	A15	RP	Blank	Blank	0,841990173	0,797156692	0,56174922	0,76739857	0,064382302
800	27	D	M	A15	A15	60	A	27	D	A15	Wound	Wound	whole	0,893270791	0,755073071	0,615320027	0,78791536	0,04891611
801	27	E	M	A45	A45	60	A	27	E	A45	LD	MeanL	Blank	0,858595967	0,74331826	0,7713269	0,782839456	0,062761246
802	27	E	M	A45	A45	60	A	27	E	A45	LP	Blank	Blank	0,840075791	0,76493299	0,778370261	0,780637637	0,060308038
803	27	E	M	A45	A45	60	A	27	E	A45	RD	MeanR	Blank	0,939265966	0,842100203	0,666735172	0,790369278	0,04479908
804	27	E	M	A45	A45	60	A	27	E	A45	RP	Blank	Blank	0,850178719	0,798172057	0,654390812	0,787725127	0,052043366
805	27	E	M	A45	A45	60	A	27	E	A45	Wound	Wound	whole	0,87998271	0,747007012	0,638438463	0,799332563	0,044541904
806	27	F	M	A30	A30	60	A	27	F	A30	LD	MeanL	Blank	0,827560782	0,77764374	0,662060678	0,780675294	0,062924364
807	27	F	M	A30	A30	60	A	27	F	A30	LP	Blank	Blank	0,88990438	0,794756353	0,863423109	0,792376196	0,046630336
808	27	F	M	A30	A30	60	A	27	F	A30	RD	MeanR	Blank	0,720152617	0,697830856	0,741082966	0,772529369	0,057952167
809	27	F	M	A30	A30	60	A	27	F	A30	RP	Blank	Blank	0,859388053	0,752107203	0,686462581	0,756144172	0,0662247
810	27	F	M	A30	A30	60	A	27	F	A30	Wound	Wound	whole	0,894559145	0,800154924	0,719089329	0,805853631	0,03695387
811	28	A	M	A30	A30	14	A	28	A	A30	LD	MeanL	Blank	0,876231372	0,807573974	0,764310062	0,658264495	0,097589698
812	28	A	M	A30	A30	14	A	28	A	A30	LP	Blank	Blank	0,77680546	0,709789872	0,90987134	0,712952769	0,069281638
813	28	A	M	A30	A30	14	A	28	A	A30	RD	MeanR	Blank	0,777541459	0,727357566	0,768401206	0,707770576	0,056942093
814	28	A	M	A30	A30	14	A	28	A	A30	RP	Blank	Blank	0,852632284	0,754378676	0,768104792	0,692591287	0,08566445
815	28	A	M	A30	A30	14	A	28	A	A30	Wound	Wound	whole	0,915783942	0,747063756	0,649901092	0,720075472	0,062511716
816	28	B	M	A15	A15	14	A	28	B	A15	LD	MeanL	Blank	0,899965405	0,778714478	0,706330299	0,696560569	0,10182946
817	28	B	M	A15	A15	14	A	28	B	A15	LP	Blank	Blank	0,808421969	0,757667661	0,78252244	0,703055942	0,092295223
818	28	B	M	A15	A15	14	A	28	B	A15	RD	MeanR	Blank	0,946974635	0,827661276	0,862972379	0,722968826	0,077348027
819	28	B	M	A15	A15	14	A	28	B	A15	RP	Blank	Blank	0,905703962	0,792673826	0,817827642	0,715780665	0,078066621

820	28	B	M	A15	A15	14	A	28	B	A15	Wound	Wound	whole	0,939559281	0,755094409	0,691603899	0,739933091	0,068674443
821	28	C	M	A45	A45	14	A	28	C	A45	LD	MeanL	Blank	0,746967614	0,687534153	0,742747545	0,725098901	0,089917397
822	28	C	M	A45	A45	14	A	28	C	A45	LP	Blank	Blank	0,845045567	0,770975351	0,768766761	0,725667775	0,087306196
823	28	C	M	A45	A45	14	A	28	C	A45	RD	MeanR	Blank	0,96622318	0,797180891	0,805764496	0,737286034	0,072987571
824	28	C	M	A45	A45	14	A	28	C	A45	RP	Blank	Blank	0,833881795	0,770482361	0,707801044	0,698881694	0,081370626
825	28	C	M	A45	A45	14	A	28	C	A45	Wound	Wound	whole	0,865219235	0,742712379	0,635770679	0,750911444	0,060464808
826	28	D	M	C	c_A30	14	C	28	D	C	LD	MeanL	Blank	0,933857381	0,818419516	0,761146426	0,731804118	0,06004156
827	28	D	M	C	c_A30	14	C	28	D	C	LP	Blank	Blank	0,946861863	0,784003913	0,666486144	0,724008518	0,058428429
828	28	D	M	C	c_A30	14	C	28	D	C	RD	MeanR	Blank	0,864472747	0,775912523	0,689453125	0,700261396	0,090211997
829	28	D	M	C	c_A30	14	C	28	D	C	RP	Blank	Blank	0,789758563	0,731935263	0,599790037	0,686159378	0,093163792
830	28	D	M	C	c_A30	14	C	28	D	C	Wound	Wound	whole	0,8979972	0,750207722	0,452624768	0,733708326	0,069965772
831	28	E	M	C	c_A15	14	C	28	E	C	LD	MeanL	Blank	0,709485292	0,680743158	0,769940376	0,705911152	0,093589487
832	28	E	M	C	c_A15	14	C	28	E	C	LP	Blank	Blank	0,800946236	0,726312816	0,844199896	0,718920521	0,076380135
833	28	E	M	C	c_A15	14	C	28	E	C	RD	MeanR	Blank	0,90910846	0,789705038	0,839649558	0,74120664	0,064456592
834	28	E	M	C	c_A15	14	C	28	E	C	RP	Blank	Blank	0,953959584	0,806648314	0,847872734	0,753163782	0,067888846
835	28	E	M	C	c_A15	14	C	28	E	C	Wound	Wound	whole	0,893655419	0,726779759	0,732800722	0,728312867	0,069645557
836	28	F	M	C	c_A45	14	C	28	F	C	LD	MeanL	Blank	0,788715124	0,733502328	0,843308151	0,753710394	0,067703559
837	28	F	M	C	c_A45	14	C	28	F	C	LP	Blank	Blank	0,938551724	0,791874349	0,75691855	0,76154154	0,059046758
838	28	F	M	C	c_A45	14	C	28	F	C	RD	MeanR	Blank	0,894920468	0,777901053	0,777744174	0,737108396	0,058348286
839	28	F	M	C	c_A45	14	C	28	F	C	RP	Blank	Blank	0,962306738	0,764340222	0,759059429	0,71791867	0,08146708
840	28	F	M	C	c_A45	14	C	28	F	C	Wound	Wound	whole	0,944447815	0,765692651	0,534790635	0,764748131	0,058315844
841	29	A	M	A15	A15	7	A	29	A	A15	LD	MeanL	Blank	0,812442958	0,773361504	0,719506741	0,676675004	0,063574807
842	29	A	M	A15	A15	7	A	29	A	A15	LP	Blank	Blank	0,79021126	0,724573672	0,74795115	0,700904333	0,063033386
843	29	A	M	A15	A15	7	A	29	A	A15	RD	MeanR	Blank	0,886632562	0,810519695	0,789559186	0,687966682	0,074250339
844	29	A	M	A15	A15	7	A	29	A	A15	RP	Blank	Blank	0,747512579	0,713269234	0,74856925	0,691047004	0,094804365
845	29	A	M	A15	A15	7	A	29	A	A15	Wound	Wound	whole	0,957460344	0,709823847	0,5097996	0,712767384	0,054704091
846	29	B	M	A30	A30	7	A	29	B	A30	LD	MeanL	Blank	0,971487463	0,806245327	0,923990011	0,682713255	0,068659628
847	29	B	M	A30	A30	7	A	29	B	A30	LP	Blank	Blank	0,880553722	0,789543688	0,841102064	0,706784663	0,064365935

848	29	B	M	A30	A30	7	A	29	B	A30	RD	MeanR	Blank	0,838608921	0,774673045	0,832415521	0,689143923	0,091311507
849	29	B	M	A30	A30	7	A	29	B	A30	RP	Blank	Blank	0,876846075	0,777965248	0,793863833	0,698480245	0,083577033
850	29	B	M	A30	A30	7	A	29	B	A30	Wound	Wound	whole	0,921299458	0,738206863	0,751918495	0,703102648	0,070737174
851	29	C	M	A45	A45	7	A	29	C	A45	LD	MeanL	Blank	0,936144888	0,81134522	0,85567224	0,697707766	0,092127466
852	29	C	M	A45	A45	7	A	29	C	A45	LP	Blank	Blank	0,831834555	0,773389995	0,873294055	0,750272511	0,064884065
853	29	C	M	A45	A45	7	A	29	C	A45	RD	MeanR	Blank	0,874932408	0,756226659	0,901931584	0,740489502	0,067067577
854	29	C	M	A45	A45	7	A	29	C	A45	RP	Blank	Blank	0,863954782	0,793259323	0,842739642	0,721419585	0,077477168
855	29	C	M	A45	A45	7	A	29	C	A45	Wound	Wound	whole	0,922536671	0,766170382	0,690249264	0,75122124	0,062004851
856	29	D	M	C	c_A15	7	C	29	D	C	LD	MeanL	Blank	0,780498624	0,729976356	0,612133265	0,710544003	0,077795739
857	29	D	M	C	c_A15	7	C	29	D	C	LP	Blank	Blank	0,839811563	0,814570904	0,752262592	0,681568894	0,079982635
858	29	D	M	C	c_A15	7	C	29	D	C	RD	MeanR	Blank	0,902684689	0,782132924	0,773123741	0,666578501	0,093358464
859	29	D	M	C	c_A15	7	C	29	D	C	RP	Blank	Blank	0,680402398	0,66892463	0,731833696	0,682037656	0,069751071
860	29	D	M	C	c_A15	7	C	29	D	C	Wound	Wound	whole	0,883138061	0,750446439	0,494209975	0,708419186	0,076002094
861	29	E	M	C	c_A30	7	C	29	E	C	LD	MeanL	Blank	0,925923824	0,848872185	0,75922662	0,678464706	0,092997816
862	29	E	M	C	c_A30	7	C	29	E	C	LP	Blank	Blank	0,881934941	0,78747642	0,867856383	0,674698333	0,063724868
863	29	E	M	C	c_A30	7	C	29	E	C	RD	MeanR	Blank	0,880147815	0,826891601	0,769899011	0,639198941	0,106420509
864	29	E	M	C	c_A30	7	C	29	E	C	RP	Blank	Blank	0,733541608	0,708046138	0,864889741	0,692020351	0,065643591
865	29	E	M	C	c_A30	7	C	29	E	C	Wound	Wound	whole	0,942849636	0,780183077	0,640844703	0,673031517	0,081822785
866	29	F	M	C	c_A45	7	C	29	F	C	LD	MeanL	Blank	0,805414677	0,749102473	0,786185503	0,72374063	0,086915582
867	29	F	M	C	c_A45	7	C	29	F	C	LP	Blank	Blank	0,779925406	0,709737062	0,915047348	0,714345931	0,080310994
868	29	F	M	C	c_A45	7	C	29	F	C	RD	MeanR	Blank	0,793663144	0,762618423	0,865425646	0,687956536	0,084508991
869	29	F	M	C	c_A45	7	C	29	F	C	RP	Blank	Blank	0,944453657	0,843873918	0,804191768	0,71813218	0,08590959
870	29	F	M	C	c_A45	7	C	29	F	C	Wound	Wound	whole	0,976108372	0,77837503	0,643639326	0,712258201	0,078492994
871	30	A	M	A45	A45	7	A	30	A	A45	LD	MeanL	Blank	0,698910236	0,672656953	0,686975956	0,661348827	0,079996292
872	30	A	M	A45	A45	7	A	30	A	A45	LP	Blank	Blank	0,665024638	0,65324086	0,738371789	0,692456529	0,073858528
873	30	A	M	A45	A45	7	A	30	A	A45	RD	MeanR	Blank	0,850108743	0,775813639	0,735843658	0,682839496	0,089075811
874	30	A	M	A45	A45	7	A	30	A	A45	RP	Blank	Blank	0,756118655	0,733614981	0,734737158	0,729559506	0,077270592
875	30	A	M	A45	A45	7	A	30	A	A45	Wound	Wound	whole	0,870225668	0,771976471	0,562296212	0,689526543	0,081384587

876	30	B	M	A15	A15	7	A	30	B	A15	LD	MeanL	Blank	0,979338527	0,823676825	0,771446705	0,677976519	0,083015559
877	30	B	M	A15	A15	7	A	30	B	A15	LP	Blank	Blank	0,797916412	0,757988095	0,787648737	0,677404671	0,078654151
878	30	B	M	A15	A15	7	A	30	B	A15	RD	MeanR	Blank	0,850878537	0,775613546	0,792703867	0,683005912	0,088516674
879	30	B	M	A15	A15	7	A	30	B	A15	RP	Blank	Blank	0,955992579	0,819598377	0,733005166	0,691099455	0,094047137
880	30	B	M	A15	A15	7	A	30	B	A15	Wound	Wound	whole	0,976473451	0,754441917	0,619237363	0,687415795	0,077747105
881	30	C	M	A30	A30	7	A	30	C	A30	LD	MeanL	Blank	0,869615555	0,79584378	0,769941986	0,708546653	0,09413895
882	30	C	M	A30	A30	7	A	30	C	A30	LP	Blank	Blank	0,815852821	0,756649554	0,78678441	0,701758298	0,087444256
883	30	C	M	A30	A30	7	A	30	C	A30	RD	MeanR	Blank	0,922573447	0,856636643	0,783934414	0,726959395	0,087776381
884	30	C	M	A30	A30	7	A	30	C	A30	RP	Blank	Blank	0,81026113	0,772978127	0,68693769	0,7465639	0,07229204
885	30	C	M	A30	A30	7	A	30	C	A30	Wound	Wound	whole	0,941861808	0,82024467	0,617765963	0,710277792	0,082054793
886	30	D	M	C	c_A45	7	C	30	D	C	LD	MeanL	Blank	0,702811956	0,67733103	0,684550524	0,710544828	0,075551743
887	30	D	M	C	c_A45	7	C	30	D	C	LP	Blank	Blank	0,836069822	0,760155976	0,811002731	0,706638312	0,081096492
888	30	D	M	C	c_A45	7	C	30	D	C	RD	MeanR	Blank	0,913176954	0,796417892	0,706441224	0,720887721	0,076885739
889	30	D	M	C	c_A45	7	C	30	D	C	RP	Blank	Blank	0,822942972	0,776484311	0,706040204	0,688799462	0,075733679
890	30	D	M	C	c_A45	7	C	30	D	C	Wound	Wound	whole	0,863435566	0,740530849	0,664360821	0,719648532	0,070724065
891	30	E	M	C	c_A15	7	C	30	E	C	LD	MeanL	Blank	0,700328708	0,664397299	0,780699968	0,709209914	0,07553685
892	30	E	M	C	c_A15	7	C	30	E	C	LP	Blank	Blank	0,741542101	0,699332952	0,846194088	0,685761955	0,080016562
893	30	E	M	C	c_A15	7	C	30	E	C	RD	MeanR	Blank	0,975832462	0,838041842	0,791974008	0,688165241	0,076319425
894	30	E	M	C	c_A15	7	C	30	E	C	RP	Blank	Blank	0,828060627	0,771329165	0,826763868	0,670538662	0,090527678
895	30	E	M	C	c_A15	7	C	30	E	C	Wound	Wound	whole	0,873222053	0,756656528	0,737776697	0,692862107	0,069981123
896	30	F	M	C	c_A30	7	C	30	F	C	LD	MeanL	Blank	0,761241376	0,715001881	0,800751925	0,741547723	0,078788909
897	30	F	M	C	c_A30	7	C	30	F	C	LP	Blank	Blank	0,878978014	0,761831284	0,877472818	0,738222672	0,07115827
898	30	F	M	C	c_A30	7	C	30	F	C	RD	MeanR	Blank	0,725314021	0,697228789	0,75488174	0,70561537	0,081704229
899	30	F	M	C	c_A30	7	C	30	F	C	RP	Blank	Blank	0,897859335	0,825863719	0,752169192	0,721596911	0,080794036
900	30	F	M	C	c_A30	7	C	30	F	C	Wound	Wound	whole	0,862480283	0,762555122	0,634889126	0,729984798	0,068249554
961	33	A	M	A45	A45	60	A	33	A	A45	LD	MeanL	Blank	0,833878398	0,778370023	0,624239266	0,661883412	0,086253418
962	33	A	M	A45	A45	60	A	33	A	A45	LP	Blank	Blank	0,79609251	0,720566094	0,734663248	0,681031995	0,089211675
963	33	A	M	A45	A45	60	A	33	A	A45	RD	MeanR	Blank	0,728919029	0,691495895	0,757139862	0,707826415	0,08395214

964	33	A	M	A45	A45	60	A	33	A	A45	RP	Blank	Blank	0,951841891	0,800033927	0,725381553	0,713910225	0,083356877
965	33	A	M	A45	A45	60	A	33	A	A45	Wound	Wound	whole	0,814509928	0,734039009	0,73209846	0,705650804	0,063451197
966	33	B	M	A15	A15	60	A	33	B	A15	LD	MeanL	Blank	0,886955619	0,779807925	0,740957022	0,662011937	0,088624147
967	33	B	M	A15	A15	60	A	33	B	A15	LP	Blank	Blank	0,875568151	0,797274292	0,805923283	0,668915664	0,081162573
968	33	B	M	A15	A15	60	A	33	B	A15	RD	MeanR	Blank	0,935958207	0,802078843	0,713511705	0,70743251	0,079210559
969	33	B	M	A15	A15	60	A	33	B	A15	RP	Blank	Blank	0,791284919	0,729055762	0,742242217	0,71571359	0,08716454
970	33	B	M	A15	A15	60	A	33	B	A15	Wound	Wound	whole	0,911493778	0,809613764	0,785445154	0,717846067	0,060396973
971	33	C	M	A30	A30	60	A	33	C	A30	LD	MeanL	Blank	0,921118319	0,822510958	0,694768965	0,701894408	0,08248411
972	33	C	M	A30	A30	60	A	33	C	A30	LP	Blank	Blank	0,910949111	0,781950116	0,917813003	0,728933391	0,063232367
973	33	C	M	A30	A30	60	A	33	C	A30	RD	MeanR	Blank	0,826145887	0,763885558	0,886539578	0,740810732	0,068647128
974	33	C	M	A30	A30	60	A	33	C	A30	RP	Blank	Blank	0,766925693	0,718146861	0,706698716	0,723090643	0,082266233
975	33	C	M	A30	A30	60	A	33	C	A30	Wound	Wound	whole	0,847952664	0,79897517	0,798631668	0,777455943	0,048297914
976	33	D	M	C	c_A45	60	C	33	D	C	LD	MeanL	Blank	0,857281804	0,79609561	0,716109395	0,715505963	0,086948484
977	33	D	M	C	c_A45	60	C	33	D	C	LP	Blank	Blank	0,9704566	0,791735172	0,691094935	0,688868936	0,085364068
978	33	D	M	C	c_A45	60	C	33	D	C	RD	MeanR	Blank	0,815016031	0,766819656	0,704133332	0,710224677	0,05730564
979	33	D	M	C	c_A45	60	C	33	D	C	RP	Blank	Blank	0,878590941	0,803561449	0,79576385	0,691348568	0,064381972
980	33	D	M	C	c_A45	60	C	33	D	C	Wound	Wound	whole	0,881828725	0,697950125	0,678667605	0,699217429	0,065917162
981	33	E	M	C	c_A15	60	C	33	E	C	LD	MeanL	Blank	0,841883719	0,762146056	0,80373776	0,723151148	0,060228464
982	33	E	M	C	c_A15	60	C	33	E	C	LP	Blank	Blank	0,719695807	0,652829885	0,857991815	0,675610774	0,083757045
983	33	E	M	C	c_A15	60	C	33	E	C	RD	MeanR	Blank					
984	33	E	M	C	c_A15	60	C	33	E	C	RP	Blank	Blank					
985	33	E	M	C	c_A15	60	C	33	E	C	Wound	Wound	whole	0,824342251	0,76112926	0,779904068	0,705596747	0,052592749
986	33	F	M	C	c_A30	60	C	33	F	C	LD	MeanL	Blank	0,830986738	0,759257674	0,663602412	0,746869736	0,0773299
987	33	F	M	C	c_A30	60	C	33	F	C	LP	Blank	Blank	0,80956316	0,709710598	0,869084418	0,768410063	0,058718651
988	33	F	M	C	c_A30	60	C	33	F	C	RD	MeanR	Blank	0,834001303	0,752599716	0,812343717	0,718110229	0,082043916
989	33	F	M	C	c_A30	60	C	33	F	C	RP	Blank	Blank	0,727851391	0,696833551	0,663248599	0,709620496	0,083136839
990	33	F	M	C	c_A30	60	C	33	F	C	Wound	Wound	whole	0,838459015	0,779947758	0,835094988	0,784694085	0,042252311
1021	35	A	M	A30	A30	14	A	35	A	A30	LD	MeanL	Blank	0,863636017	0,784304142	0,748027444	0,684791129	0,07104205

1022	35	A	M	A30	A30	14	A	35	A	A30	LP	Blank	Blank	0,854457974	0,751763821	0,740112662	0,710452355	0,069278802
1023	35	A	M	A30	A30	14	A	35	A	A30	RD	MeanR	Blank	0,841241837	0,745810866	0,836631894	0,707565115	0,075136413
1024	35	A	M	A30	A30	14	A	35	A	A30	RP	Blank	Blank	0,866097987	0,779099882	0,821249127	0,716178445	0,075249194
1025	35	A	M	A30	A30	14	A	35	A	A30	Wound	Wound	whole	0,973927379	0,762161911	0,587662756	0,720587423	0,057451556
1026	35	B	M	A45	A45	14	A	35	B	A45	LD	MeanL	Blank					
1027	35	B	M	A45	A45	14	A	35	B	A45	LP	Blank	Blank	0,961269677	0,824721575	0,73499006	0,657802525	0,059394205
1028	35	B	M	A45	A45	14	A	35	B	A45	RD	MeanR	Blank	0,903405607	0,770541489	0,854971409	0,704856623	0,056643287
1029	35	B	M	A45	A45	14	A	35	B	A45	RP	Blank	Blank	0,887070894	0,77084285	0,835913897	0,737735597	0,063637205
1030	35	B	M	A45	A45	14	A	35	B	A45	Wound	Wound	whole	0,883637309	0,757076144	0,528621078	0,7218309	0,051879361
1031	35	C	M	A15	A15	14	A	35	C	A15	LD	MeanL	Blank	0,910492659	0,763173938	0,843477428	0,726534091	0,070992825
1032	35	C	M	A15	A15	14	A	35	C	A15	LP	Blank	Blank	0,868435085	0,771808922	0,887076735	0,750745179	0,05022198
1033	35	C	M	A15	A15	14	A	35	C	A15	RD	MeanR	Blank	0,9605667	0,820978045	0,868741035	0,735323626	0,062371148
1034	35	C	M	A15	A15	14	A	35	C	A15	RP	Blank	Blank	0,904141426	0,768666089	0,846193492	0,757956683	0,06175226
1035	35	C	M	A15	A15	14	A	35	C	A15	Wound	Wound	whole	0,948753536	0,765022695	0,539619267	0,740663845	0,042487347
1036	35	D	M	C	c_A30	14	C	35	D	C	LD	MeanL	Blank	0,809208333	0,757246137	0,728801429	0,701813717	0,091555112
1037	35	D	M	C	c_A30	14	C	35	D	C	LP	Blank	Blank	0,854336619	0,730198026	0,871517599	0,728676364	0,064554825
1038	35	D	M	C	c_A30	14	C	35	D	C	RD	MeanR	Blank	0,866217613	0,805761933	0,743114948	0,70272854	0,052886997
1039	35	D	M	C	c_A30	14	C	35	D	C	RP	Blank	Blank	0,936071157	0,817513406	0,864584565	0,678065588	0,070810703
1040	35	D	M	C	c_A30	14	C	35	D	C	Wound	Wound	whole	0,880349398	0,752083242	0,613587797	0,731509484	0,041036539
1041	35	E	M	C	c_A45	14	C	35	E	C	LD	MeanL	Blank	0,946466684	0,788210869	0,751317918	0,74812909	0,076894022
1042	35	E	M	C	c_A45	14	C	35	E	C	LP	Blank	Blank	0,830414295	0,725375473	0,818075836	0,719579219	0,079385684
1043	35	E	M	C	c_A45	14	C	35	E	C	RD	MeanR	Blank	0,900749505	0,76309967	0,867834151	0,71425231	0,066313931
1044	35	E	M	C	c_A45	14	C	35	E	C	RP	Blank	Blank	0,942211628	0,768531859	0,884432495	0,715010103	0,066055855
1045	35	E	M	C	c_A45	14	C	35	E	C	Wound	Wound	whole	0,962548256	0,801619351	0,755555511	0,735325483	0,054064629
1046	35	F	M	C	c_A15	14	C	35	F	C	LD	MeanL	Blank	0,862786114	0,766282856	0,819084942	0,763425516	0,06403205
1047	35	F	M	C	c_A15	14	C	35	F	C	LP	Blank	Blank	0,96986717	0,81154722	0,869654119	0,754126215	0,063490904
1048	35	F	M	C	c_A15	14	C	35	F	C	RD	MeanR	Blank	0,972778261	0,796446264	0,899501383	0,751110759	0,059974545
1049	35	F	M	C	c_A15	14	C	35	F	C	RP	Blank	Blank	0,865128815	0,76463604	0,854813457	0,735484824	0,068047312

1050	35	F	M	C	c_A15	14	C	35	F	C	Wound	Wound	whole	0,944120288	0,771418273	0,762021363	0,764580517	0,051359836
1081	37	A	M	C	c_A30	14	C	37	A	C	LD	MeanL	Blank	0,876694798	0,797198117	0,666470289	0,738347841	0,064998976
1082	37	A	M	C	c_A30	14	C	37	A	C	LP	Blank	Blank	0,778765321	0,740829825	0,760800779	0,744550593	0,054970772
1083	37	A	M	C	c_A30	14	C	37	A	C	RD	MeanR	Blank	0,845318317	0,765797317	0,640413105	0,765446447	0,057729766
1084	37	A	M	C	c_A30	14	C	37	A	C	RP	Blank	Blank	0,860240638	0,782414615	0,488354117	0,755940502	0,06693693
1085	37	A	M	C	c_A30	14	C	37	A	C	Wound	Wound	whole	0,957688451	0,76453948	0,552975774	0,727650956	0,0539215
1086	37	B	M	C	c_A15	14	C	37	B	C	LD	MeanL	Blank	0,707825422	0,593032479	0,155202001	0,74840393	0,070277645
1087	37	B	M	C	c_A15	14	C	37	B	C	LP	Blank	Blank	0,544745505	0,538042724	0,151704758	0,819514357	0,039627775
1088	37	B	M	C	c_A15	14	C	37	B	C	RD	MeanR	Blank	0,372775555	0,366932929	0,043239716	0,811652889	0,038601976
1089	37	B	M	C	c_A15	14	C	37	B	C	RP	Blank	Blank	0	2,64049E-05	0,000372178	0,955555556	0,020123927
1090	37	B	M	C	c_A15	14	C	37	B	C	Wound	Wound	whole	0,795155406	0,729691923	0,588566065	0,781817307	0,05489564
1091	37	C	M	C	c_A45	14	C	37	C	C	LD	MeanL	Blank	0,938628674	0,798948944	0,796639383	0,76958401	0,044205208
1092	37	C	M	C	c_A45	14	C	37	C	C	LP	Blank	Blank	0,755462825	0,727782786	0,614463508	0,769303145	0,044500502
1093	37	C	M	C	c_A45	14	C	37	C	C	RD	MeanR	Blank	0,8677513	0,78189379	0,733461559	0,751920617	0,058554954
1094	37	C	M	C	c_A45	14	C	37	C	C	RP	Blank	Blank	0,825841308	0,693605363	0,651236951	0,752476214	0,068669031
1095	37	C	M	C	c_A45	14	C	37	C	C	Wound	Wound	whole	0,883453965	0,716256022	0,463594764	0,784937154	0,045659965
1096	37	D	M	A30	A30	14	A	37	D	A30	LD	MeanL	Blank	0,818524003	0,74722904	0,680065691	0,772857983	0,058935762
1097	37	D	M	A30	A30	14	A	37	D	A30	LP	Blank	Blank	0,889545202	0,79752636	0,809607327	0,757549463	0,050895639
1098	37	D	M	A30	A30	14	A	37	D	A30	RD	MeanR	Blank	0,833652794	0,761879921	0,782673478	0,724679656	0,044476575
1099	37	D	M	A30	A30	14	A	37	D	A30	RP	Blank	Blank	0,952114522	0,817320466	0,669705749	0,753826761	0,06555539
1100	37	D	M	A30	A30	14	A	37	D	A30	Wound	Wound	whole	0,871035755	0,793165624	0,641198337	0,759953336	0,043684285
1101	37	E	M	A15	A15	14	A	37	E	A15	LD	MeanL	Blank	0,902387559	0,780768633	0,534374237	0,746156693	0,071109757
1102	37	E	M	A15	A15	14	A	37	E	A15	LP	Blank	Blank	0,817869782	0,747173548	0,685471416	0,744533244	0,068009885
1103	37	E	M	A15	A15	14	A	37	E	A15	RD	MeanR	Blank	0,874170661	0,750810564	0,668712735	0,72778199	0,090151051
1104	37	E	M	A15	A15	14	A	37	E	A15	RP	Blank	Blank	0,719320178	0,690026879	0,672961414	0,756651744	0,065795332
1105	37	E	M	A15	A15	14	A	37	E	A15	Wound	Wound	whole	0,931237996	0,733515978	0,490402043	0,729771118	0,077968388
1106	37	F	M	A45	A45	14	A	37	F	A45	LD	MeanL	Blank	0,85638839	0,780237615	0,62966156	0,738580015	0,078164265
1107	37	F	M	A45	A45	14	A	37	F	A45	LP	Blank	Blank	0,86376828	0,737413406	0,732384086	0,740538166	0,071979644

1108	37	F	M	A45	A45	14	A	37	F	A45	RD	MeanR	Blank	0,803435564	0,772847772	0,711411893	0,748231981	0,051295844
1109	37	F	M	A45	A45	14	A	37	F	A45	RP	Blank	Blank	0,769541502	0,745219707	0,541629374	0,751163053	0,049861846
1110	37	F	M	A45	A45	14	A	37	F	A45	Wound	Wound	whole	0,857933462	0,74205029	0,561256051	0,768440585	0,051036053
1321	45	A	M	A15	A15	7	A	45	A	A15	LD	MeanL	Blank	0,849102318	0,785992384	0,773058653	0,721946646	0,067891156
1322	45	A	M	A15	A15	7	A	45	A	A15	LP	Blank	Blank	0,878490329	0,80873245	0,644670725	0,744444198	0,053084342
1323	45	A	M	A15	A15	7	A	45	A	A15	RD	MeanR	Blank	0,755897164	0,696971536	0,792323291	0,711585168	0,077616237
1324	45	A	M	A15	A15	7	A	45	A	A15	RP	Blank	Blank	0,857187986	0,772483706	0,811772943	0,757682201	0,066017409
1325	45	A	M	A15	A15	7	A	45	A	A15	Wound	Wound	whole	0,88327986	0,726191878	0,59861207	0,742897441	0,057193191
1326	45	B	M	A45	A45	7	A	45	B	A45	LD	MeanL	Blank	0,798181236	0,750007093	0,515403628	0,737497736	0,053220332
1327	45	B	M	A45	A45	7	A	45	B	A45	LP	Blank	Blank	0,789241612	0,763579667	0,672551811	0,722819099	0,073181823
1328	45	B	M	A45	A45	7	A	45	B	A45	RD	MeanR	Blank	0,832718253	0,768649876	0,826805353	0,754277859	0,063568633
1329	45	B	M	A45	A45	7	A	45	B	A45	RP	Blank	Blank	0,715785742	0,678628147	0,796911538	0,768899791	0,067878835
1330	45	B	M	A45	A45	7	A	45	B	A45	Wound	Wound	whole	0,88988024	0,777817726	0,672662795	0,760386231	0,062167276
1331	45	C	M	A30	A30	7	A	45	C	A30	LD	MeanL	Blank	0,83807683	0,780906141	0,778847933	0,769028777	0,062673637
1332	45	C	M	A30	A30	7	A	45	C	A30	LP	Blank	Blank	0,825929523	0,75651598	0,874107063	0,766683207	0,067438668
1333	45	C	M	A30	A30	7	A	45	C	A30	RD	MeanR	Blank	0,885362327	0,786208928	0,806286037	0,771943032	0,064495342
1334	45	C	M	A30	A30	7	A	45	C	A30	RP	Blank	Blank	0,870213032	0,760754168	0,806133151	0,791592435	0,060318917
1335	45	C	M	A30	A30	7	A	45	C	A30	Wound	Wound	whole	0,951196671	0,781474113	0,706085026	0,778774014	0,057254027
1336	45	D	M	C	c_A15	7	C	45	D	C	LD	MeanL	Blank	0,85106498	0,795404613	0,785192788	0,740877677	0,074705347
1337	45	D	M	C	c_A15	7	C	45	D	C	LP	Blank	Blank	0,82762593	0,770675004	0,755042672	0,732053752	0,070113189
1338	45	D	M	C	c_A15	7	C	45	D	C	RD	MeanR	Blank	0,865939736	0,814611018	0,597442627	0,747379573	0,054794868
1339	45	D	M	C	c_A15	7	C	45	D	C	RP	Blank	Blank	0,723178387	0,702512681	0,794652104	0,726084393	0,0642455
1340	45	D	M	C	c_A15	7	C	45	D	C	Wound	Wound	whole	0,888536036	0,773966312	0,598776042	0,748277459	0,057498995
1341	45	E	M	C	c_A45	7	C	45	E	C	LD	MeanL	Blank	0,91419369	0,779842675	0,768318951	0,778813386	0,061112619
1342	45	E	M	C	c_A45	7	C	45	E	C	LP	Blank	Blank	0,881528616	0,739134312	0,783201158	0,752193554	0,058878123
1343	45	E	M	C	c_A45	7	C	45	E	C	RD	MeanR	Blank	0,797027349	0,752043724	0,757953286	0,719062145	0,065241107
1344	45	E	M	C	c_A45	7	C	45	E	C	RP	Blank	Blank	0,82032299	0,780021846	0,754721403	0,715921912	0,063204485
1345	45	E	M	C	c_A45	7	C	45	E	C	Wound	Wound	whole	0,861477256	0,743779659	0,698203802	0,743777088	0,06076725

1346	45	F	M	C	c_A30	7	C	45	F	C	LD	MeanL	Blank	0,877407074	0,810649157	0,727028131	0,793665857	0,055215532
1347	45	F	M	C	c_A30	7	C	45	F	C	LP	Blank	Blank	0,796999037	0,732361376	0,712940156	0,769807867	0,062475599
1348	45	F	M	C	c_A30	7	C	45	F	C	RD	MeanR	Blank	0,818207204	0,720766962	0,741380036	0,747858033	0,081664099
1349	45	F	M	C	c_A30	7	C	45	F	C	RP	Blank	Blank	0,838723898	0,718964517	0,753588021	0,768524702	0,062564783
1350	45	F	M	C	c_A30	7	C	45	F	C	Wound	Wound	whole	0,818409026	0,74432528	0,649852514	0,778163851	0,06072347
1351	46	A	M	C	c_A45	7	C	46	A	C	LD	MeanL	Blank	0,883922458	0,779641807	0,683697999	0,688745943	0,097179293
1352	46	A	M	C	c_A45	7	C	46	A	C	LP	Blank	Blank	0,951531589	0,828440487	0,641522706	0,688076084	0,078926259
1353	46	A	M	C	c_A45	7	C	46	A	C	RD	MeanR	Blank	0,836384237	0,794374585	0,721154392	0,68313963	0,09385506
1354	46	A	M	C	c_A45	7	C	46	A	C	RP	Blank	Blank	0,724676967	0,686510682	0,745257914	0,697748318	0,082245127
1355	46	A	M	C	c_A45	7	C	46	A	C	Wound	Wound	whole	0,878186464	0,756034791	0,570916533	0,697050972	0,08057997
1356	46	B	M	C	c_A15	7	C	46	B	C	LD	MeanL	Blank	0,917333901	0,817774296	0,777679503	0,66263156	0,089333993
1357	46	B	M	C	c_A15	7	C	46	B	C	LP	Blank	Blank	0,764494359	0,745268881	0,653004646	0,673713098	0,084406476
1358	46	B	M	C	c_A15	7	C	46	B	C	RD	MeanR	Blank	0,87383008	0,776216745	0,75363785	0,704524605	0,080558399
1359	46	B	M	C	c_A15	7	C	46	B	C	RP	Blank	Blank	0,852987707	0,782745838	0,826025546	0,718392742	0,077644773
1360	46	B	M	C	c_A15	7	C	46	B	C	Wound	Wound	whole	0,902064562	0,774305284	0,640115023	0,704065564	0,080443456
1361	46	C	M	C	c_A30	7	C	46	C	C	LD	MeanL	Blank	0,887533784	0,803383768	0,668069005	0,664826233	0,082375141
1362	46	C	M	C	c_A30	7	C	46	C	C	LP	Blank	Blank	0,829692662	0,778706908	0,712970018	0,694874941	0,081468126
1363	46	C	M	C	c_A30	7	C	46	C	C	RD	MeanR	Blank	0,90358144	0,82577008	0,771960378	0,678023768	0,083091323
1364	46	C	M	C	c_A30	7	C	46	C	C	RP	Blank	Blank	0,79101789	0,746860385	0,821650207	0,703258551	0,07945764
1365	46	C	M	C	c_A30	7	C	46	C	C	Wound	Wound	whole	0,951228201	0,826179385	0,648267269	0,67387603	0,083250168
1366	46	D	M	A45	A45	7	A	46	D	A45	LD	MeanL	Blank	0,840082347	0,783508599	0,713231146	0,708884282	0,098059364
1367	46	D	M	A45	A45	7	A	46	D	A45	LP	Blank	Blank	0,906341076	0,80703795	0,778372586	0,688230125	0,091986693
1368	46	D	M	A45	A45	7	A	46	D	A45	RD	MeanR	Blank	0,862488747	0,758016109	0,623230636	0,691141578	0,077273986
1369	46	D	M	A45	A45	7	A	46	D	A45	RP	Blank	Blank	0,903632939	0,817725301	0,714318991	0,689479874	0,076125695
1370	46	D	M	A45	A45	7	A	46	D	A45	Wound	Wound	whole	0,888406813	0,734251916	0,619712055	0,702240751	0,074369162
1371	46	E	M	A15	A15	7	A	46	E	A15	LD	MeanL	Blank	0,943855882	0,84305203	0,827524364	0,716796091	0,07658481
1372	46	E	M	A15	A15	7	A	46	E	A15	LP	Blank	Blank	0,81672442	0,704311311	0,862714767	0,700881606	0,075424892
1373	46	E	M	A15	A15	7	A	46	E	A15	RD	MeanR	Blank	0,917936385	0,791254342	0,263375431	0,684608798	0,086575952

1374	46	E	M	A15	A15	7	A	46	E	A15	RP	Blank	Blank	0,875912428	0,801419854	0,792760372	0,678669414	0,084751876
1375	46	E	M	A15	A15	7	A	46	E	A15	Wound	Wound	whole	0,899111092	0,749153793	0,715111494	0,710853792	0,07946608
1376	46	F	M	A30	A30	7	A	46	F	A30	LD	MeanL	Blank	0,845147729	0,774920225	0,811792493	0,648665019	0,09360647
1377	46	F	M	A30	A30	7	A	46	F	A30	LP	Blank	Blank	0,940798759	0,810440183	0,787995815	0,675606009	0,091930242
1378	46	F	M	A30	A30	7	A	46	F	A30	RD	MeanR	Blank	0,74494803	0,712395847	0,814427435	0,668885773	0,083252097
1379	46	F	M	A30	A30	7	A	46	F	A30	RP	Blank	Blank	0,878742993	0,787381709	0,751671433	0,63778355	0,098323599
1380	46	F	M	A30	A30	7	A	46	F	A30	Wound	Wound	whole	0,912873626	0,756368101	0,682027578	0,65923961	0,082188437
1381	47	A	M	C	c_A15	60	C	47	A	C	LD	MeanL	Blank	0,853985488	0,802887738	0,712589741	0,705471274	0,085523307
1382	47	A	M	C	c_A15	60	C	47	A	C	LP	Blank	Blank	0,941019297	0,821142435	0,770288289	0,706482876	0,075920127
1383	47	A	M	C	c_A15	60	C	47	A	C	RD	MeanR	Blank	0,970243573	0,808891058	0,724424243	0,708046294	0,089656736
1384	47	A	M	C	c_A15	60	C	47	A	C	RP	Blank	Blank	0,831180573	0,786874473	0,784685194	0,723318143	0,082501635
1385	47	A	M	C	c_A15	60	C	47	A	C	Wound	Wound	whole	0,950605154	0,787191033	0,748463869	0,721565815	0,066320731
1386	47	B	M	C	c_A30	60	C	47	B	C	LD	MeanL	Blank	0,867248774	0,755762637	0,66129446	0,678620584	0,089181386
1387	47	B	M	C	c_A30	60	C	47	B	C	LP	Blank	Blank	0,869061053	0,743863821	0,728920162	0,679615624	0,092284009
1388	47	B	M	C	c_A30	60	C	47	B	C	RD	MeanR	Blank	0,85811305	0,783158243	0,738714159	0,680738588	0,087843501
1389	47	B	M	C	c_A30	60	C	47	B	C	RP	Blank	Blank	0,847595632	0,771586895	0,772643387	0,703633072	0,089457402
1390	47	B	M	C	c_A30	60	C	47	B	C	Wound	Wound	whole	0,943938017	0,792506278	0,78417629	0,692023851	0,066964964
1391	47	C	M	C	c_A45	60	C	47	C	C	LD	MeanL	Blank	0,862036586	0,787510931	0,911416352	0,718562983	0,049952077
1392	47	C	M	C	c_A45	60	C	47	C	C	LP	Blank	Blank	0,888267815	0,814799726	0,941402733	0,724005319	0,048098414
1393	47	C	M	C	c_A45	60	C	47	C	C	RD	MeanR	Blank	0,74448204	0,688498318	0,720578849	0,710481715	0,083094391
1394	47	C	M	C	c_A45	60	C	47	C	C	RP	Blank	Blank	0,754565477	0,718342423	0,748403013	0,754259234	0,074600704
1395	47	C	M	C	c_A45	60	C	47	C	C	Wound	Wound	whole	0,823387563	0,780419707	0,852808714	0,732416194	0,046424939
1396	47	D	M	A15	A15	60	A	47	D	A15	LD	MeanL	Blank	0,876025915	0,805166006	0,795193255	0,73417927	0,068761577
1397	47	D	M	A15	A15	60	A	47	D	A15	LP	Blank	Blank	0,899168074	0,826267123	0,762509882	0,708299944	0,095164353
1398	47	D	M	A15	A15	60	A	47	D	A15	RD	MeanR	Blank	0,918705881	0,817197382	0,710388899	0,708102325	0,084434768
1399	47	D	M	A15	A15	60	A	47	D	A15	RP	Blank	Blank	0,851466238	0,773829341	0,717036545	0,704985233	0,088715051
1400	47	D	M	A15	A15	60	A	47	D	A15	Wound	Wound	whole	0,963237405	0,795234144	0,770807683	0,719628947	0,069424909
1401	47	E	M	A30	A30	60	A	47	E	A30	LD	MeanL	Blank	0,892393827	0,79489392	0,784854174	0,719343167	0,086227551

1402	47	E	M	A30	A30	60	A	47	E	A30	LP	Blank	Blank	0,832054734	0,736261308	0,780548155	0,704129305	0,087253569
1403	47	E	M	A30	A30	60	A	47	E	A30	RD	MeanR	Blank	0,8846246	0,767455876	0,835519493	0,734318959	0,083967721
1404	47	E	M	A30	A30	60	A	47	E	A30	RP	Blank	Blank	0,854309618	0,785119355	0,731091857	0,675753885	0,10065056
1405	47	E	M	A30	A30	60	A	47	E	A30	Wound	Wound	whole	0,860821545	0,799803019	0,822676718	0,717274746	0,065695633
1406	47	F	M	A45	A45	60	A	47	F	A45	LD	MeanL	Blank	0,8080616	0,739807308	0,868087709	0,732705399	0,072904225
1407	47	F	M	A45	A45	60	A	47	F	A45	LP	Blank	Blank	0,916403294	0,802358627	0,879945993	0,724562924	0,07696161
1408	47	F	M	A45	A45	60	A	47	F	A45	RD	MeanR	Blank	0,870724618	0,740516543	0,790655732	0,75340756	0,071185545
1409	47	F	M	A45	A45	60	A	47	F	A45	RP	Blank	Blank	0,768412411	0,707861662	0,690986991	0,744517638	0,069903162
1410	47	F	M	A45	A45	60	A	47	F	A45	Wound	Wound	whole	0,971222878	0,839714229	0,796874523	0,774013879	0,048133662
